# A mathematics for medicine: The Network Effect

**DOI:** 10.3389/fphys.2014.00456

**Published:** 2014-12-09

**Authors:** Bruce J. West

**Affiliations:** Mathematics and Information Science Directorate, Army Research OfficeResearch Triangle Park, NC, USA

**Keywords:** physiologic networks, statistical extrema, complexity and disease, fractional calculus, complexity hypothesis

## Abstract

The theory of medicine and its complement *systems biology* are intended to explain the workings of the large number of mutually interdependent complex physiologic networks in the human body and to apply that understanding to maintaining the functions for which nature designed them. Therefore, when what had originally been made as a simplifying assumption or a working hypothesis becomes foundational to understanding the operation of physiologic networks it is in the best interests of science to replace or at least update that assumption. The replacement process requires, among other things, an evaluation of how the new hypothesis affects modern day understanding of medical science. This paper identifies linear dynamics and Normal statistics as being such arcane assumptions and explores some implications of their retirement. Specifically we explore replacing Normal with fractal statistics and examine how the latter are related to non-linear dynamics and chaos theory. The observed ubiquity of inverse power laws in physiology entails the need for a new calculus, one that describes the dynamics of fractional phenomena and captures the fractal properties of the statistics of physiological time series. We identify these properties as a necessary consequence of the complexity resulting from the network dynamics and refer to them collectively as The Network Effect.

## 1. Introduction

As scientists we measure things; the position of stars in the sky, the amount of rainfall in a region over the year or the number of heart beats per minute of a patient in our care. These numbers tell us about the phenomena we want to understand. An astronomer deduces whether the faint dot over head is a fixed star, a moving planet or a rocketing comet using the physical theories of cosmology. The numbers tell the meteorologist if there is a pattern of increasing or decreasing rainfall and whether that pattern indicates an organized change in the weather. A physician determines whether the pattern in the heart beats reveals if the patient has a cardiovascular problem that requires intervention or s/he is having an anxiety attack. Each science organizes measurements in ways that communicate the most to the practitioner and taken as a group they constitute the scientific view of the world, which is to say that if it is a matter of science it can be measured.

In keeping with the data-based perspective, the measurements in and theories of molecular biology and genetics in the latter half of the twentieth century produced a shift in medical outlook from the pathologies of the cardiovascular, respiratory and motor control networks to the influence of molecules on health and well being. However, in the past decade or so the pendulum has begun to swing back from the concentration on individual molecules to a focus on the properties of networks of molecules and a determination of the emergent properties of such complex interactive networks. This is particularly true in physiological networks, which, as Bashan et al. (2012) point out, under neural regulation exhibit complex, non-stationary, intermittent, scale-invariant and non-linear behavior.

Herein we do not trace the various mechanisms (or the development of mathematical models) that produce scale-free microscopic (Barabasi and Oltvai, 2004) or macroscopic (Newman, 2010) networks, since such efforts exist in large number. Instead we examine the properties of physiologic time series and identify those properties that emerge from the underlying network dynamics. In particular we focus on the inverse power-law (IPL) statistics that result from the temporal complexity generated by network dynamics, separate and distinct from the topological complexity that is the result of the connectivity of such networks, see for example Turalska et al. (2009), West et al. (2014) and references therein. Note that there is probably some nomenclature adopted in this paper that is not familiar and therefore a Glossary is provided in Appendix 6.1 for handy reference.

In medicine it is often the outlier that determines the outcome of an intervention because its influence on the process may be irreversible as, for example, in the damage caused by a heart attack. In complex phenomena these extrema have recently been called “black swans,” a name coined by Taleb (2007), and this name has captured the imagination of the scientific and lay communities alike. Black swans are the unpredictable extrema that lead to flash crashes, bursts and catastrophic failures in health care systems (West and Clancy, 2010), physical phenomena such as earthquakes and wildfires (Sahs et al., 2012); medical phenomena such as epileptic seizures (Osorio et al., 2010) and cardiac mortality (Hayano et al., 2011); economic systems such as the financial market (Taleb, 2007), and so on.

These outliers have also been called “dragon kings” by Sornette (1998, 2009). The dragon kings are distinguished from black swans in that dragon kings are assumed to not share the statistics of the other data in the process but have an unrelated, possibly deterministic, mechanism for their generation. Consequently, it has been argued that such dragon kings can be predictable even though black swans cannot. However, we are less interested in the names of these various extrema than we are in how they can be used to distinguish among various phenomena.

Then there is the question of how long one must wait for the occurrence of these outliers. In statistics it is argued that the average waiting time for an event of a given size to occur is proportional to the inverse of the probability of that event occurring (Gumbel, 1954). Consequently, the lower the probability of an event the longer the wait. Thus, we should have to wait a very long time for a black swan or dragon king—but is that true in real world data, particularly in medicine? The answer to that question appears to be that although rare these extrema occur much more frequently than one would predict for a process with Normal statistics. Herein we present a calculus that is able to take such a distinction into account and form a new hypothesis concerning the nature of the statistics of physiological time series data and what they entail.

### 1.1. Law of errors

The present essay addresses those elusive patterns in the medical data that distinguish between the healthy and pathological and how such patterns might be pinned down in order to describe the properties of complex medical phenomena. From a brief historical review it will become apparent that the working assumption that the statistics of physiologic time series are Normal, an assumption made in the nineteenth century, has become the implicit and often the explicit foundation for much of the formal theory of medicine. Here we motivate abandoning this assumption and initiate the exploration of a theory entailed by formulating a new hypothesis based on the insights into complexity made in the past few decades.

Complexity was first dealt with in the rapidly developing physics of the eighteenth century by recognizing that no experiment ever gives the same result twice. No matter how skilled the experimenter, how carefully the experiment is prepared, how precise the instruments used, an experiment repeated *N* times and prepared in “exactly” the same way each time will yield *N* different numbers. Scientists in the seventeenth and eighteenth centuries struggled with the best way to characterize the scatter in the data associated with this ensemble of measurements from ostensibly the same experiment, see for example West and Grigolini (2011) for a somewhat more extended discussion.

A breakthrough in empiricism was made at the end of the eighteenth century by introducing the arithmetic average of *N* measurements *X*_1_, *X*_2_, …, *X_N_*:

(1) $$\bar{X}=\frac{1}{N}\underset{k=1}{\sum^{N}}X_{k}$$

as “the” way to characterize an ensemble of measurements. This was accompanied by introducing an arbitrary measure of the quality of the average as a way to characterize the scattered data, that being the deviation of the second moment from the square of the average:

(2) $$\sigma^{2}\equiv \frac{1}{N}\underset{k=1}{\sum^{N}}X_{k}^{2}-{\bar{X}}^{2},$$

the variance, or its square-root the standard deviation σ. This treatment of data was published independently in 1809 by the German polymath Johann Carl Friedrich (Gauss, 1809) and the American mathematician (Adrian, 1809) both of whom provided the bell-shaped curve as the representation of the Normal distribution of statistical variability. This approach evolved into the Law of Frequency of Errors as a consequence of the proof of the Central Limit Theorem provided a year later by Laplace (1810).

The Law of Errors is an interesting name for the variability in experimental data since it implies that the average is the “proper” description of the data and deviations from that value constitute errors. Consequently the Law of Errors and the Normal distribution are consistent with the mechanical laws of motion formulated by Newton. These laws of motion predict certain outcomes for experiments; the measurements of those outcomes reveal a variation about the predicted value and the Normal distribution gives the degree of variability in the measurements about the predicted or average value. The narrower the bell-shaped curve the closer the measured value is to the predicted one. Put simply, the Normal distribution implies that there is a right answer to the question being experimentally asked. Thus, even through no theory of medicine existed at the time the average value was assumed to provide the predication of that theory if and when it would be formulated.

Many phenomena are described by the Normal distribution or Normal statistics, but it is often forgotten that the measurement error in such phenomena must satisfy the four criteria of the Central Limit Theorem in order to be Normally distributed. Expressed in the language of the Law of Errors these criteria are: (1) the errors are independent; the error in a given experiment does not depend on the error in any other experiment; (2) the errors are additive; the total error made is the sum of the separate errors; (3) the statistics of each error is the same; the statistical process producing the error does not change from experiment to experiment; and (4) the width of the distribution is finite; the standard deviation converges to a finite value as the number of measured errors increases. These criteria are recorded here because complex medical phenomena often violate one or more of these conditions necessary to satisfy the mathematical proof of the Central Limit Theorem and it is here that the real world deviates from the expected variability imposed by Normal statistics (West, 2006).

There is a wide array of medical phenomena that manifest Normal statistics including height (Quetelet, 1835), birth weight (O'Cathain et al., 2002), body temperature (Mackowiak et al., 1992), and the logarithm of blink rate (Bentivoglio et al., 1997). On the other hand, there is an even greater list of non-Normal statistical medical phenomena including heart rate variability (Peng et al., 1993), neuronal avalanches (Plenz, 2012), interbreath and interstride interval variability (West, 2006), to name a few. It is the latter physiologic phenomena that we are interested in understanding and this requires the study of how the statistical properties modify our understanding. Of particular importance is our understanding of extrema and how these extrema change with increasing variability of the underlying statistics.

### 1.2. The network effect

Recently the identification of emergent phenomena across multiple disciplines, from the swarming of insects (Yates et al., 2009), the schooling of fish (Katz et al., 2011) and the flocking of birds (Cavagna et al., 2010) observed in animal groups by naturalists; to the spatiotemporal activity of the brain (Beggs and Plenz, 2003, Fraiman et al., 2009, Chialvo, 2010) observed by neurophysiologists; to the collective and cooperative behavior observed in social groups studied by psychologists and sociologists; all demonstrate collective behavior reminiscent of particle dynamics near the critical phase transitions studied by physicists (Stanley, 1971). Each of these disciplines has demonstrated the need to investigate the dynamics of complex networks across scales in order to develop a deeper understanding of how large-scale behavior emerges from microscale dynamics and the sensitivity of the observed behavior to those dynamics.

Of particular interest to us here are the medical fields in which we observe a need for a system wide approach (Richardson and Goldstein, 2010). The recent discoveries in medicine were propelled by the successes of molecular biology and genetics that have made available genomic blueprints of numerous organisms, which are complemented by extensive experimental data describing cell functions. At the same time however the realization came that biological function emerges out of the interaction of numerous molecular components, making the detailed knowledge of specific components at any level of organization insufficient to capture macroscopic functionality. One example of this limitation that we return to subsequently is the study of the cardiovascular systems in order to understand, predict and ultimately modify (in order to heal) cardiac function.

Despite experimental developments, the ability of science to make theoretical predictions of the behavior of complex networks is still in its infancy. The adoption of methods from non-equilibrium statistical physics have demonstrated limitations, resulting from the fact that living networks, in contrast to inert physical materials, are extremely heterogeneous, non-generic, highly specialized and operate far from an equilibrium state (Elsasser, 1981). We hypothesize that “The Network Effect” (TNE) is to impose a level of complexity that eludes analytic dynamic descriptions based on systems of ordinary and/or stochastic differential equations, as well as the equivalent partial differential equations describing the phase space evolution of probability density functions (PDF's).

Herein we demonstrate that what was for a very long time a niche branch of mathematics, the fractional calculus, might very well be able to span the gap between the inert materials of physics and the living networks of medicine. Consequently TNE may well be summarized as the need for a system of fractional differential equations to describe the dynamics of complex networks. The support for this hypothesis is primarily empirical as we show, with the exception of the connection established by West et al. (2014) for a model non-linear dynamic network.

Although developed along side the classical calculus, fractional differential equations have only recently been shown to be a convenient way to describe the dynamics of complex phenomena characterized by long-term memory and spatial heterogeneity (Podlubny, 1999, West et al., 2003). Fractional differential equations have been demonstrated to capture the time evolution of fractal processes, such as in anomalous diffusion, viscoelasticity and turbulent fluid flow, as reviewed by West and Grigolini (2011). In spite of the success of the mathematical descriptions of such processes there has been a lack of identification and interpretation of mechanisms that entail fractional dynamic equations in the context of complex physiological networks. Herein we suggest how this barrier might be either overcome or circumvented.

### 1.3. Statistics of extrema

An extreme event may be thought of as the occurrence of an incident which in some phenomenon exhibits itself outside the typical region of fluctuation as measured by some appropriately chosen variable—wind gust loads on airplanes in flight, the highest temperatures or lowest pressures in meteorology, floods, and droughts in hydrology, and human life spans, all fall in this category. A perhaps even longer list of phenomena of medical importance, including heart attacks, the falling of the elderly, epileptic seizures, traumatic brain injury, could be drawn up. To be adequately prepared for such occurrences knowledge of the underlying statistical behavior of such events must be in hand.

In 1935 Emil Gumbel derived an expression for a PDF of the maxima in data sets (Gumbel, 1954, Reiss and Thomas, 1997)

(3) P(x)=exp[−exp[−χ(x−μ)]];−∞≤x≤∞,

where μ is related to the mean and χ to the standard deviation of the extrema variable *x*. In the original derivation of this cumulative distribution the variable was the oldest ages in human life spans. Subsequently, the Gumbel PDF was applied to other extreme events, such as those cited above. The derivation of the PDF was based on the two assumptions: (1) given a random variable *X*, such as the height of a river or the magnitude of an earthquake, the successive measurements are statistically independent of one another; (2) the statistics are stationary in time, that is, the PDF is independent of an overall shift in time. The domain of attraction of this PDF encompasses most of the commonly used distributions, that is, PDF's for which there exists a mean and standard deviation such that as the number of data points becomes arbitrarily large, the limit of the extrema PDF approaches the Gumbel form.

**Figure 1:**
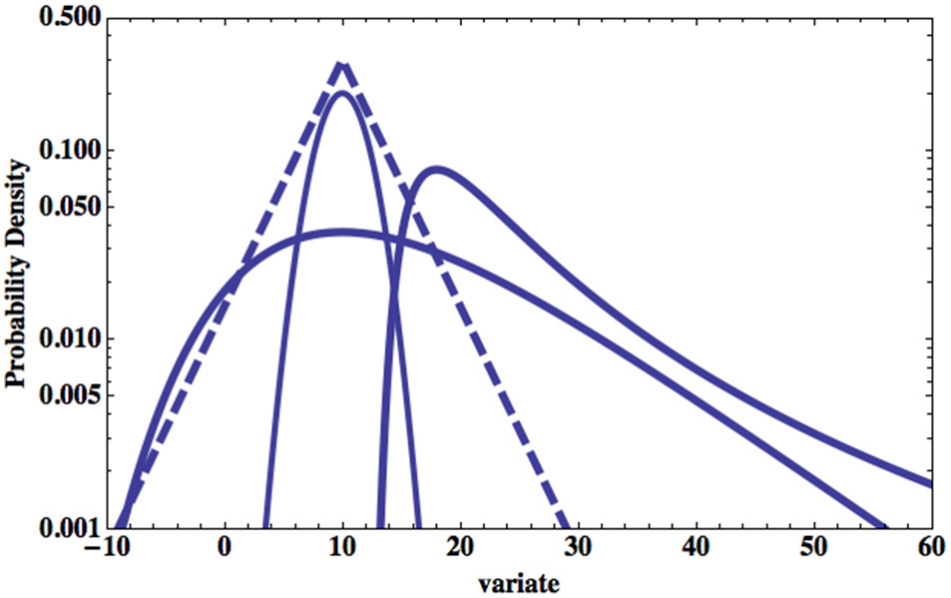
**Four PDF's are plotted on log-linear graph paper**. The parabolic solid curve is a Normal PDF and the dashed is a Laplace PDF. The broader of the two remaining distributions is the Gumbel PDF given by Equation (4). The remaining extrema distribution is that of Fréchet (1927) and is discussed subsequently.

Figure 1 compares the Normal and the Laplace (1810) PDF's to that of Gumbel as well as to a second type of extreme value PDF due to Fréchet (1927). Note that a PDF is obtained from the negative derivative of the probability so that for the Gumbel PDF we obtain from Equation (3)

(4) $$p(x)=-\frac{dP(x)}{dx}=\chi \exp\left[-\chi \left(x-\mu \right)\right]\exp\left[-\exp\left[-\chi \left(x-\mu \right)\right]\right].$$

It is evident from the figure where Equation (4) is plotted that the large excursion of the variate from the central values dominate the extrema PDF's.

Of particular interest to us here are the extreme properties of fluctuations in medical observables. If the maximum fluctuation in body temperature doubles an individual may be in some distress but if the maximum interval between heart beats double the pathology may be life threatening. Part of the reason for this difference can be traced to the changes in the behavior of extrema properties for a Normal statistical process and non-Normal processes, say one described by Pareto, i.e., IPL statistics. Extrema in stock market fluctuations, Taleb's black swans Taleb (2007), generated by an underlying Pareto distribution are unpredictable and have a distribution that does not approach the Gumbel form, but does converge on the Fréchet form. On the other hand, these extrema could also be Sornette's dragon kings (Sornette, 2009) when they are transiently organized into extreme events that are statistically or perhaps mechanically different from those in the underlying distribution that produces the smaller values of the fluctuations. An example of the latter mechanism was worked out in detail by Montroll and Shlesinger (1984) who leverage the moments of a distribution with finite central moments using a renormalization group argument to generate the Pareto tail in the income PDF's for Western society independently of the initial distribution. We present this argument subsequently.

The lack of interchangeability of black swans and dragon kings in the interpretation of medical pathologies has not yet been addressed explicitly, as we subsequently discuss. This distinction becomes important in a medical context given the recent progress by de Souza Cavalcante et al. (2013) in real time forecasting of an impending extreme event (“dragon king”), but more importantly that it is demonstrably possible to perturb the system to suppress the onset of the extrema for certain chaotic mechanisms.

### 1.4. Preview

In Section 2 the idea is developed that the PDF's generated by complex phenomena, particularly those in medicine, require a new method of quantification; one based on fractal statistics, self-similarity, and the fractional calculus. Some renormalization group scaling ideas are taken from Zaslavsky (2002) but other sources for the mathematical infrastructure developed are also discussed. The renormalization group properties of the statistical distribution are discussed, including the existence of complex fractal dimensions that explain the empirical harmonic modulation of the scaling observed in some data. Thus, information in fractal phenomena is coupled across multiple scales, as for example, observed in the architecture of the mammalian lung (West et al., 1986, Nelson et al., 1990, Weibel, 2000); manifest in the long-range correlations in human gait (Hausdorff et al., 1996, West, 1999); measured in the human cardiovascular network (Peng et al., 1993) and observed in a number of other physiologic contexts (West et al., 2008). These all appear to be consequences of TNE.

Fractal processes are dynamically rich in interconnected scales with no one scale or set of scales dominating. The solutions to the fractional equations of motion for a category of such processes are given in Section 3 using scaling arguments. The alpha-stable Lévy distribution is one such solution that has been suggested to describe the statistics of heart rate variability (HRV) of both healthy and diseased individuals (Peng et al., 1993). A discussion of these statistics is given in Section 4 where the fractional calculus is shown to provide a truncated Lévy distribution to describe healthy individuals. It is argued that the truncation is determined by a physiological control process that is suppressed in individuals that suffer a cardiac induced death. Extreme value theory is used to distinguish between black swans and dragon kings using the length of time one would wait before the reoccurrence of an event of a given magnitude. This might translate into how long one can survive with a critical illness given the time one has already survived with that illness.

In Section 5 we present some conclusions based on the application of the fractional calculus and scaling to physiological time series and discuss the implications of the TNE hypothesis.

## 2. Fractional calculus

Physics has the most complete description of the dynamics of phenomena, from the deterministic force laws of Newton to the stochastic equations of Langevin (1908). By comparison medicine lacks the foundational principles necessary to generate such mathematical descriptions, whether in terms of the equations for the dynamic variables or the phase space equations for the corresponding PDF's This limitation is due in large part to the complexity of medical phenomena and the lack of formal understanding of that complexity. Herein we provide a glimpse into a strategy for how this limitation may be overcome.

The inherent complexity of physiologic structure, dynamics and function was captured in the latter part of the last century through the concept of fractals. The inventor, developer and champion of this idea was the late Mandelbrot (1977) who discussed the anatomical structure of the lung and the convoluted surface of the mammalian brain in terms of fractal geometry. Of course he did not restrict himself to static objects but introduced the notion of fractal statistics to describe intermittent stochastic phenomena. What tied the geometrical and statistical fractals together was the idea of scaling and much of the past quarter century was devoted to finding the scaling behavior of time series for heart beats, breathing intervals, stride intervals, and many other phenomena identified as belonging to the study of fractal physiology (West, 2010). A crucial point about these studies is that by identifying a time series by means of a fractal function *f*(*t*) immediately disqualifies the traditional ordinary differential equations as a way of describing its dynamics since the ordinary derivative of a fractal function diverges. Consequently, it became necessary to show that a fractional derivative of a fractal function is another fractal function implying that the fractional calculus is the appropriate way to describe the dynamics of fractal phenomena (West et al., 2003, West and Clancy, 2010, West and Grigolini, 2011).

In this section we show that the scaling behavior of the fractional kinetic equation (FKE) entails the notion of complex fractal dimensions. The real part of the complex dimension is the IPL index and the imaginary part yields a periodic modulation of the IPL distribution. The existence of such a complex dimension is sketched out using data from a number of physiologic phenomena.

### 2.1. Fractional kinetics equation

In this section we introduce the kinetic equation for the evolution of the probability *P*(*x, t*)*dx* of a dynamic process *X*(*t*) along a fractal trajectory having a value in the interval (*x*, *x* + *dx*) at time *t*. Zaslavsky (2002) provides an excellent description of the relation between chaotic trajectories generated by non-linear equations of motion and the fractional kinetics of the PDF. He considers the infinitesimal changes of *P*(*x, t*) in time along chaotic trajectories whose local averages yield a FKE, which is to say an equation of motion for the PDF in phase space in terms of fractional derivatives. There are a number of alternative derivations of the FKE including the continuous time random walk (CTRW) of Montroll and Weiss (1965) as reviewed by Metzler and Klafter (2000); the extension of the CTRW using subordination (Gorenflo et al., 2007); as well as the fractional generalized Langevin equation, see for example, Lutz (2012), West and Grigolini (2011). One of the simplest form of the FKE is

(5) $$\partial_{t}^{\alpha }\left[P(x,t)\right]=K_{\beta }\partial_{\left|x\right|}^{\beta }\left[P(x,t)\right]$$

and the non-integer parameters α and β are scaling exponents that characterize the fractal structure of the trajectories in Zaslavsky's approach and consequently yield fractional derivatives in time and space, respectively. Of course it is also possible that the diffusion coefficient is dependent on *t* and/or *x*, but we do not consider these cases here and instead refer the reader back to the literature (Zaslavsky, 2002, Klafter et al., 2012). It remains for us to interpret the symbols indicating the fractional derivatives in time and in space, which we do in Section 3.

Equation (5) is sometimes called the fractional Fokker-Planck equation (FFPE) with zero potential because it can be generalized by introducing a potential function in complete analogy with the historical Fokker-Planck equation. When α = 1 Equation (5) reduces to the anomalous diffusion equation (Seshadri and West, 1982, Metzler and Klafter, 2000, West et al., 2003)

(6) $$\frac{\partial P(x,t)}{\partial t}=K_{\beta }\partial_{\left|x\right|}^{\beta }\left[P(x,t)\right].$$

Here the anomaly arises from the heterogeneity in the phase space variable captured through the non-local character of the spatial fractional derivative. It is not necessary to review the fractional calculus in order to understand the solutions to Equation (5) or (6); the understanding required for our purposes can be achieved from their scaling properties.

### 2.2. Renormalization group scaling

Zaslavsky (2002) applied a renormalization group (RG) transformation to the system dynamics such that the scaling properties of the incremental changes in space and time are

(7) $$R:\;\Delta x\to \lambda_{x}\Delta x\;,\;\Delta t\to \lambda_{T}\Delta t$$

which apply after some averaging in a restricted space-time domain and (λ_*x*_, λ_*T*_) are scaling parameters. He goes on to say that a basic feature of renormalization group kinetics is that the FKE is invariant under the operation of a renormalization group transformation *R*{·}:

(8) $$R\left\{\partial_{t}^{\alpha }\left[P(x,t)\right]\right\}=R\left\{K_{\beta }\partial_{\left|x\right|}^{\beta }\left[P(x,t)\right]\right\}\;$$

implying that the FKE satisfies the scaling behavior

(9) $$\lambda_{T}^{\alpha }\partial_{t}^{\alpha }\left[P(x,t)\right]=\lambda_{x}^{\beta }K_{\beta }\partial_{\left|x\right|}^{\beta }\left[P(x,t)\right].$$

The scaling results from the fractional powers of the differentials in the basic definition of the fractional derivative.

This renormalization procedure may be applied an arbitrary number of times to the FKE and consequently the resulting fractional differential equation remains valid only if the ratio of the scaling parameters satisfies

(10) $$\underset{n\to \infty }{\lim}{\left(\frac{\lambda_{x}^{\beta }}{\lambda_{T}^{\alpha }}\right)}^{n}=1.$$

The FKE is linear so that the sum of the individual fixed-point solutions is also a solution. In this way the fixed point equation Equation (10) has an infinite number of solutions

(11) $$\frac{\lambda_{x}^{\beta }}{\lambda_{T}^{\alpha }}=e^{in2\pi }\;;\;n=0,\;\pm 1,\;\pm 2,\ldots$$

where the fixed point solutions have integer *n*. Consequently, indexing the time parameter with the fixed point index the ratio of the time to space parameters becomes

(12) $$\frac{\alpha_{n}}{\beta }=\frac{\ln\lambda_{x}}{\ln\lambda_{T}}+i\frac{2\pi n}{\beta \ln\lambda_{T}}\;;\;\alpha_{0}=\alpha .$$

Indexing the time parameter by *n* is arbitrary and is only intended to distinguish among the various fixed point solutions to the FKE.

In Section 3 we show that the solution to Equation (5) has the general scaling form

(13) $$P(x,t)=\frac{1}{t^{\delta }}F\left(\frac{x}{t^{\delta }}\right);\delta =\alpha /\beta ,$$

using the zero-order solution to Equation (12). Consequently the first moment of the dynamic variable takes the form

(14) $$\begin{array}{l} \langle x;t\rangle =\int xP(x,t)dx=\int \frac{x}{t^{\delta }}F\left(\frac{x}{t^{\delta }}\right)dx\\ \;=D_{\alpha \beta }^{\left(0\right)}t^{\delta } \end{array}$$

where the coefficient can be calculated to be Zaslavsky (2002)

(15) $$D_{\alpha \beta }^{\left(0\right)}=\int yF(y)dy=K_{\beta }\Gamma \left(1+\beta \right).$$

However, if we include the higher-order fixed point solutions from Equation (12) into the average we obtain

(16) $$\langle x;t\rangle =t^{\delta }\underset{n=0}{\sum^{\infty }}A_{n}\cos\left(2\pi n\frac{\ln t}{\ln\lambda_{T}}\right)$$

resulting in periodic variations in the average value, with variations in ln *t*, the logarithm of time for time series data, having period ln λ_*T*_.

The expansion coefficients *A*_*n*_ in Equation (16) can be explicitly calculated when the kinetic equation of motion has a discrete renormalization invariance, see, for example, Hanson et al. (1985), Montroll and Shlesinger (1984), West et al. (1986). The lowest-order coefficient is given by *A*_0_ = *D*^(0)^_αβ_. On the other hand, these coefficients can also be used heuristically as expansion coefficients much like what is done with empirical Fourier series fitting the expansion coefficients to time series data.

### 2.3. Complex fractal dimensions

It is important to note that the concept of fractal dimension implies that the underlying process is self-similar, but the fact that the dimension is complex implies even richer properties of the process. The real part of the complex dimension yields the inverse power-law index for the Pareto distribution. The time series data is seen to have bursts of activity followed by long quiescent intervals during which no event occurs. However, when one of these bursts is examined with higher resolution it is seen to be composed of bursts separated by relatively long quiescent intervals. The relative amount of bursting and resting is apportioned by the scaling index. The imaginary part of the dimension indicates that there is a memory superposed on the Pareto statistics producing log-periodic modulations of the moments. It is useful to understand that this apparently bizarre phenomenon is not new.

Over 40 years ago Novikov (1966) in his investigation of the properties of turbulent fluid flow discovered the periodically modulated scaling properties of intermittent stochastic processes. He considered a general Poisson process supplemented by “nested” pulses of activity. The power spectral density function was consequently determined to satisfy a scaling relation of the renormalization group form and the predicted spectrum was a modulated IPL. Novikov's prediction regarding the log-periodicity in turbulence has subsequently been observed by Zhou and Sornette (2002). Thus, whether the functional form of Equation (16) arises in the study of the moments of a processes, or in its spectrum, it indicates a process that is void of a characteristic scale and has long-range correlations induced by nested bursts of activity as also pointed out by West and Fan (1993).

**Figure 2:**
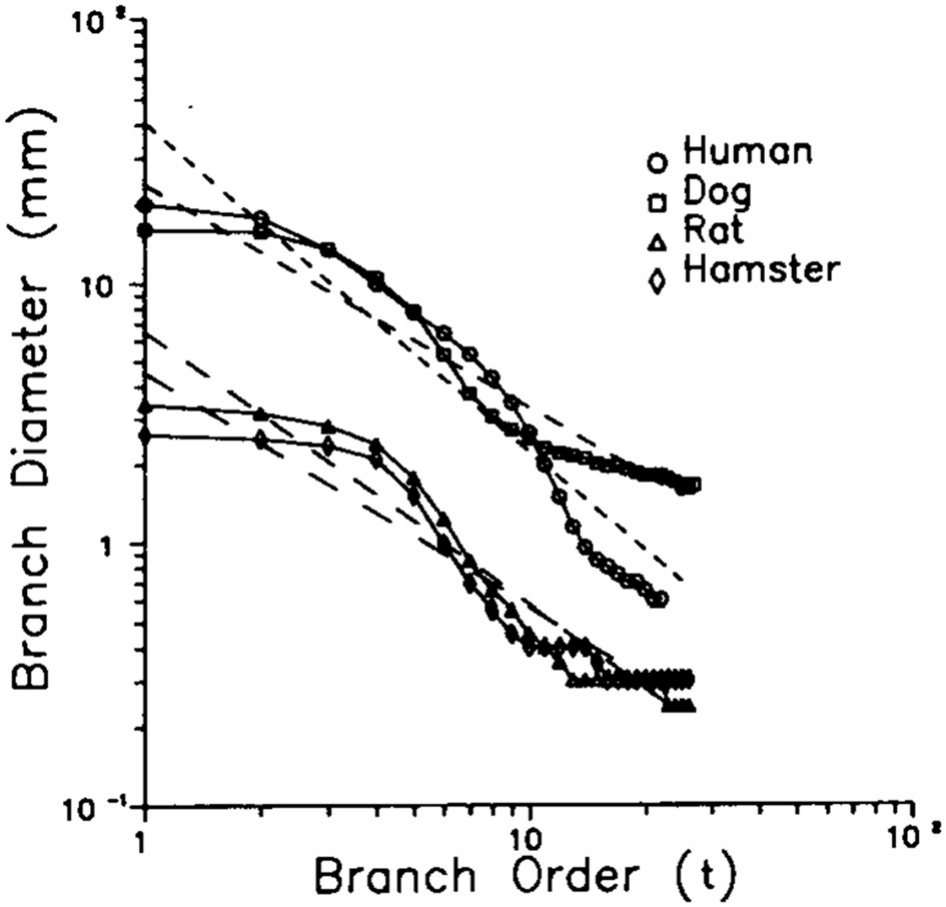
**The data from Raabe et al. (1976) for the average diameter of the mamallian lung for four distinct species is compared with the predictions of the fractal model of the lung West et al. (1986)**. The symbols are the data points and the solid curves the results of the fractal model. The parameter values yield the slopes δ = 1.26 (humans), 1.05 (rats), 0.86 (dog), and 0.90 (hamster), with periods *ln*λ_*T*_ = 2.20 for humans and rats; *ln*λ_*T*_ = 2.40 for dogs and hamsters (From Shlesinger and West, 1991 with permission).

The log-periodic variability of an observable average was first discussed in a physiologic context by Shlesinger and West (1991) to describe the scaling behavior of the bronchial airway network of the mammalian lung. In the application to physiology the solution Equation (16) takes the heuristic form

(17) $$\langle x;t\rangle =\frac{1}{t^{\delta }}\left[A_{0}+A_{1}\cos\left(2\pi \frac{\ln t}{\ln\lambda_{T}}\right)\right]$$

where 〈*x*; *t*〉 is the average diameter of the bronchial airway at generation *t*, *A*_0_ and *A*_1_ are empirical constants, and the generation *t* denotes the number of branchings of the bronchial tree starting from the trachea *t* = 0. Consequently, a result of TNE on the bronchial network is that it is characterized by having a complex fractal dimension

(18) $$D=\delta +i\frac{2\pi }{\ln\lambda_{T}}$$

whose imaginary part produces the modulation of the dominant IPL behavior of the average diameter. This periodic modulation is clearly seen in Figure 2 for four distinct species, human, dog, rat, and hamster for all generations and is apparently a general property of mammalian species and is called the fractal lung model (West et al., 1986).

**Figure 3:**
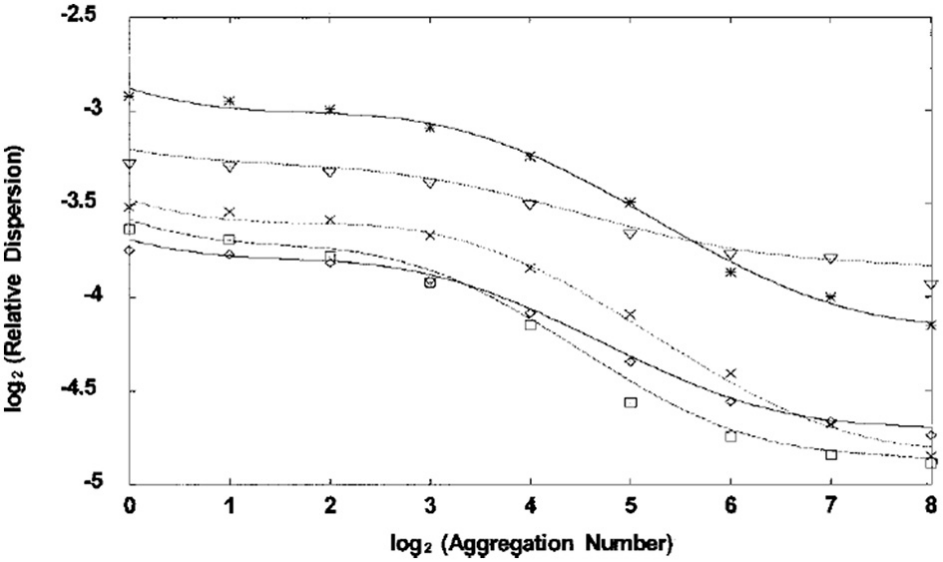
**The logarithm base 2 of the relative dispersion is plotted vs. the exponent of 2 in the numbers of aggregated data points for each of six subjects**. Each time series is 2 h long and consists of between seven and eight thousand data points. The lines are the best fits to the data and it is clear that the curves describe modulated inverse power laws (From West et al., 1999 with permission).

The log-periodic modulation of the IPL behavior of the average diameter in the bronchial airway network is only one of many such phenomenological regularities observed in physiology. Another is cerebral blood flow (CBF) velocity measured using transcranial Doppler ultrasonography, which is not strictly constant. West et al. (1999) use the dimensionless relative dispersion, the ratio of the standard deviation to the mean, to show by systematically aggregating the data that the correlations in the beat-to-beat CBF time series data is the modulated IPL depicted in Figure 3. This scaling of the CBF time series indicates the existence of long-time memory in the underlying control process. They argued that allometric control (West and Grigolini, 2010) enables the CBF to maintain a relatively constant perfusion.

The CBF time series for 2 h of data from each of six subjects is processed to obtain the relative dispersions depicted in Figure 3. The processing procedure is to first calculate the relative dispersion using all the data as indicated by the zero point on the horizontal axis in the figure. Next each of the neighboring data points is added together to obtain half the original number of data points and the relative dispersion is calculated again. This is the 1 indicated on the horizontal axis in the figure. These data points are aggregated again in the same way and the relative dispersion is calculated a third time to obtain point 2. This process is repeated six more times with the data and plotted as shown. For each subject the relative dispersion is successively aggregated in this way and plotted against the size of the aggregation and is seen to yield a modulated IPL PDF. The underlying theory on which this processing technique is based is given by Bassingthwaighte et al. (1994) for fractal time series where the relative dispersion is shown to satisfy a renormalization group relation.

**Figure 4:**
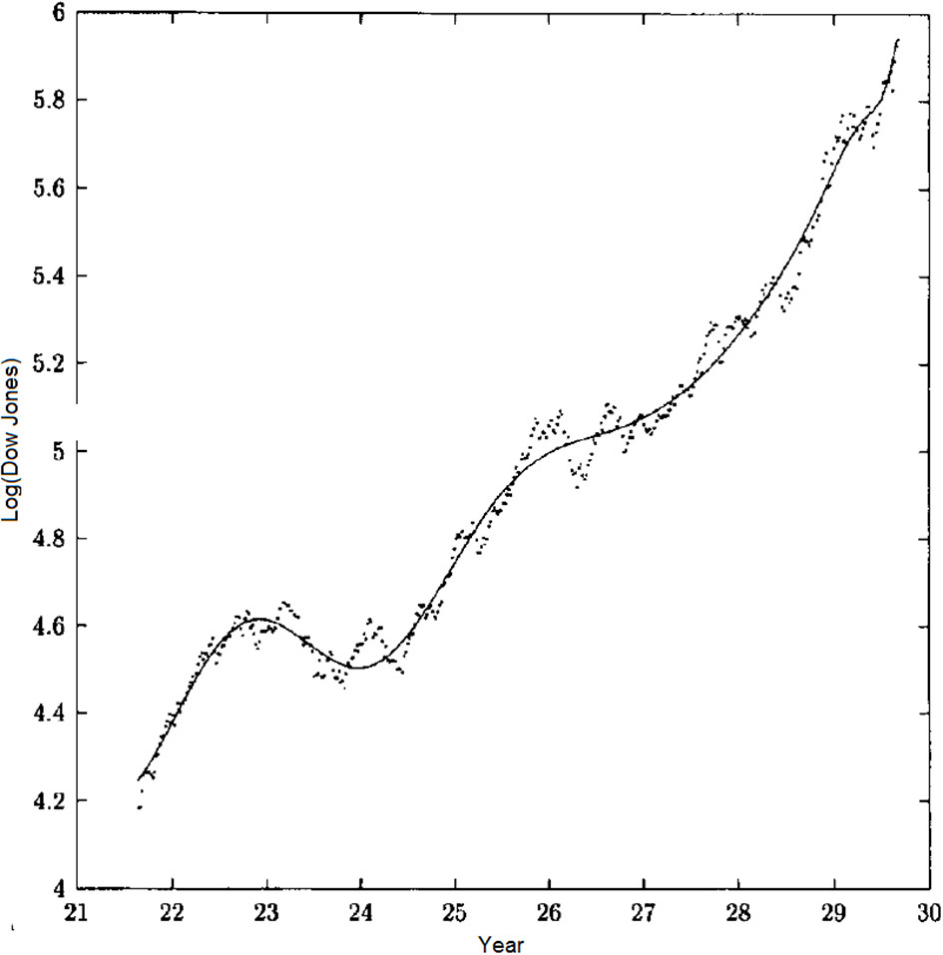
**The time dependence of the logarithm of the Dow Jones stock exchange index from June 1921 to September 1929 and the best fit of the parameters using the log-periodic modulated solution to the renormalization group relation**. (From Sornette and Johansen, 1997 with permission).

These are only two examples of physiologic phenomena with complex fractal dimensions that are revealed through modulation of the processed time series data as a consequence of the TNE. Sornette (1998) also emphasized the log-periodic corrections to scaling produced by the imaginary part of the complex fractal dimension in his extensive discussion of the concept of discrete scale invariance. He Sornette and Johansen (1997) implemented the renormalization group idea to postdict stock market crashes, that is to “predict” historical stock market crashes, fitting the log-periodic modulation of the solution to the renormalization group relation to historical data. This distinctive modulation is obtained using the Dow Jones time series financial data for the United States stock market crash of 1929 depicted in Figure 4. The solid line segment is the RG solution fit to the data. Predicting the occurrence of a crash from historical stock market time series subsequently became equivalent to predicting the arrival of a dragon king (Sornette and Johansen, 1997).

This analysis suggest the possible use of extrema in the prediction of mortality as we subsequently discuss.

## 3. Solutions to fractional kinetic equations

The method for obtaining the PDF solution to the FKE with fractional derivatives in both space and time has been presented elsewhere West and West (2012), but for the sake of completeness I sketch out the method here as well. So now we interpret the symbols for the fractional derivatives in Equation (5): ∂^α^_*t*_[·] is the Caputo fractional derivative in time (Caputo, 2001) defined below in terms of Laplace transforms; ∂^β^_|*x*|_[·] is the Reisz-Feller fractional derivative (Feller, 1968) in one space dimension defined in terms of Fourier transforms and *K*_β_ is a generalized diffusion coefficient. The most direct explicit definition of the solutions to the fractional differential equations of motion is in terms of their combined Fourier-Laplace transforms, which we now present.

### 3.1. The Mittag-Leffler function

The Fourier transform of the symmetric Reisz-Feller operator ∂^β^_|*x*|_[·] acting on an analytic function *f(x)* is as shown in Appendix 6.2 (Seshadri and West, 1982, Metzler and Klafter, 2000):

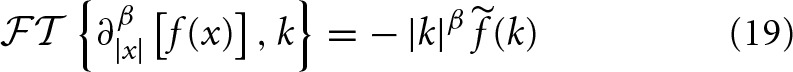



where $\tilde{f}$(*k*) is the Fourier transform of *f*(*x*). The Laplace transform of a Caputo fractional time derivative ∂^α^_*t*_[·] acting on the analytic function *g*(*t*) is

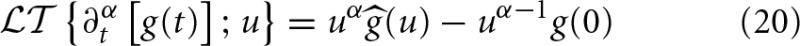



where $\hat{g}$(*u*) is the Laplace transform of *g*(*t*) and *g*(0) is its initial value. Consequently the phase space dynamics given by Equation (5) can be expressed as the joint Fourier-Laplace transform

(21) $$u^{\alpha }P^{*}(k,u)-u^{\alpha -1}=-K_{\beta }{\left|k\right|}^{\beta }P^{*}(k,u)$$

and the asterisk denotes the double transform and we have used the normalization condition on the characteristic function in the form $\tilde{P}$(*k* = 0, *t*) = 1. Therefore, after some rearrangement of terms in Equation (21) the solution in Fourier-Laplace space can be written

(22) $$P^{*}(k,u)=\frac{u^{\alpha -1}}{u^{\alpha }+K_{\beta }{\left|k\right|}^{\beta }}.$$

The PDF that solves the FKE is given by the inverse Fourier-Laplace transform of Equation (22).

We note that the space-time representation of the solution to the FKE for various combinations of α and β and potential functions are reviewed by Metzler and Klafter (2000), who show how to derive Equation (5) using the CTRW of Montroll and Weiss (1965).

The inverse Laplace transform of the Fourier-Laplace solution to the FKE yields the characteristic function in terms of a function first obtained by the mathematician Mittag-Leffler at the turn of the twentieth century:

(23) $$\tilde{P}(k,t)=E_{\alpha }\left(-K_{\beta }{\left|k\right|}^{\beta }t^{\alpha }\right)$$

in terms of the infinite series that now bears his name

(24) $$E_{\alpha }\left(z\right)\equiv \underset{n=0}{\sum^{\infty }}\frac{z^{n}}{\Gamma \left(n\alpha +1\right)}.$$

The time dependence of the Mittag-Leffler function (MLF) is extremely interesting. At early times the MLF has the analytic form of the stretched exponential (West et al., 2003):

(25) $$\underset{t\to 0}{\lim}E_{\alpha }\left(-\lambda t^{\alpha }\right)=\exp\left[-\frac{\lambda t^{\alpha }}{\Gamma \left(\alpha +1\right)}\right];$$

at late time it has the analytic form of an inverse power law (West et al., 2003):

(26) $$\underset{t\to \infty }{\lim}E_{\alpha }\left(-\lambda t^{\alpha }\right)=\frac{1}{\Gamma \left(1-\alpha \right)\lambda t^{\alpha }},$$

and the analytic series smoothly joins these two asymptotic expressions. Consequently, the relatively benign Poisson statistics at α = 1, where the MLF reduces to an exponential, becomes the intermittent IPL statistics for 0 < α < 1. The complexity of the resulting statistics is captured in the IPL index much like the allometry exponent captures the complexity of fractal structure of allometric phenomena (West and West, 2012).

### 3.2. Scaling solution

Uchaikin (2000) inverse transformed Equation (22) for arbitrary α and β to obtain the analytic form for the PDF; but that level of mathematical detail is not necessary for the present discussion. The desired insight is provided by utilizing the scaling properties of the characteristic function Equation (23) and considering the PDF in the form of the inverse Fourier transform

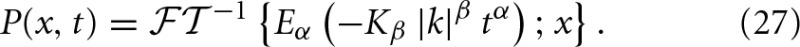



The series expansion for the MLF given by Equation (24) allows us to write the scaling relation

(28) $$P(\lambda_{x}x,\lambda_{T}t)=\underset{n=0}{\sum^{\infty }}\frac{{\left(-K_{\beta }\lambda_{T}^{\alpha }t^{\alpha }\right)}^{n}}{\Gamma \left(n\alpha +1\right)}\cdot \frac{\Gamma \left(n\beta +1\right)}{{\left|\lambda_{x}x\right|}^{n\beta +1}}$$

where the second factor in the summation is the result of applying the Tauberian Theorem (Zygmund, 1935) to the inverse Fourier transform of |*k*|^*n*β^. A renormalization group scaling equation emerges when the parameters satisfy the equality λ_*x*_ = λ^α/β^_*T*_, which is the lowest-order fixed-point solution to Equation (12), resulting in Equation (28) reducing to

(29) $$P(\lambda_{T}^{\alpha /\beta }x,\lambda_{T}t)=\frac{1}{\lambda_{T}^{\alpha /\beta }}P(x,t).$$

If we now select the time parameter to be λ_*T*_ = 1/*t*, so that in Equation (29) the scaled variable *x*/*t*^α/β^ becomes the new dynamic variable we can write

(30) $$P(x,t)=\frac{1}{t^{\alpha /\beta }}P\left(\frac{x}{t^{\alpha /\beta }},1\right).$$

Finally, the PDF that solves the FKE in terms of the similarity variable *x*/*t*^μ_*x*_^ satisfies the scaling equation

(31) $$P(x,t)=\frac{1}{t^{\mu_{x}}}F_{x}\left(\frac{x}{t^{\mu_{x}}}\right)\text{ and }\mu_{\text{x}}=\alpha /\beta ,$$

as introduced in Equation (13).

The function *F*_*x*_(·) in Equation (31) is left unspecified but it is analytic in the similarity variable *x*/*t*^μ_*x*_^. In a standard diffusion process *X*(*t*) is the displacement of a diffusing particle from its initial position at time *t*, and for vanishing small dissipation the scaling parameter is μ_*x*_ = 1/2 since α = 1, β = 2 and the functional form of *F*_*x*_(·) is a Gauss distribution. However, for general complex phenomenon there is a broad class of PDF's for which the functional form of *F*_*x*_(·) is not Gaussian and the scaling index μ_*x*_ ≠ 1/2. For example, the stable Lévy process Montroll and West (1979), Samorodnitsky and Taqqu (1994), Seshadri and West (1982), Zolotarev (1986) scales in this way and the Lévy index is in the range 0 < ϑ ≤ 2, with the equality holding for the Gauss distribution and the scaling index in Equation (31) is related to the Lévy index by μ_*x*_ = 1/ϑ.

### 3.3. Lévy statistics

In the previous section we noted that a process described by an alpha-stable Lévy distribution satisfies the general scaling solution to the FKE. More explicitly we note that the Fourier transform of a PDF is the characteristic function for a process. Consequently, the inverse Fourier transform of the characteristic function given by Equation (23) yields the PDF

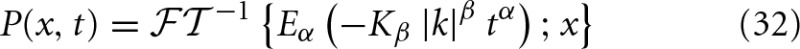



When α = 1 we know that the MLF reduces to an exponential in which case the PDF is the alpha-stable Lévy distribution in space *L*_β_(·) with a Lévy index β and a “width” that increases linearly with time:

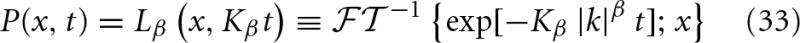



The series representation for the Lévy distribution is given in a number of places, see for example Montroll and West (1979):

(34) $$\begin{array}{l} L_{\beta }\left(x,K_{\beta }t\right)=\underset{n=1}{\sum^{\infty }}\frac{{\left(-1\right)}^{n+1}\Gamma \left[\beta n+1\right]\sin\left[\beta n\pi /2\right]}{\pi \Gamma \left(n\right)}\frac{1}{\left|x\right|}{\left(\frac{K_{\beta }t}{{\left|x\right|}^{\beta }}\right)}^{n};\\ \;-\infty <x<\infty \end{array}$$

whose lowest-order term is the IPL

(35) $$\underset{\left|x\right|\to \infty }{\lim}L_{\beta }\left(x,K_{\beta }t\right)=\frac{\Gamma \left[1+\beta \right]\sin\left[\beta \pi /2\right]}{\pi }\frac{K_{\beta }t}{{\left|x\right|}^{\beta +1}}.$$

Note that in the discussion up to this point we have interpreted *x* as the “space” variable, but this choice of words was used to facilitate the presentation. In fact *X*(*t*) has been called the dynamic variable, variate or random variable of interest and *x* is the corresponding phase space variable. The variate could be the time interval between successive heart beats and *t* would then be the chronological time and the clock is started when the first of these beats occurred. Consequently both the random variable and the independent variable would be measures of time. For example, if the heart rate variability (HRV) were given by a Lévy distribution then *x* in Equation (34) would be the interbeat time interval and *L*_β_(*x, K*_β_*t*) dx would be the probability that the interbeat interval falls in the interval (*x*, *x* + *dx*) at time *t*.

**Figure 5:**
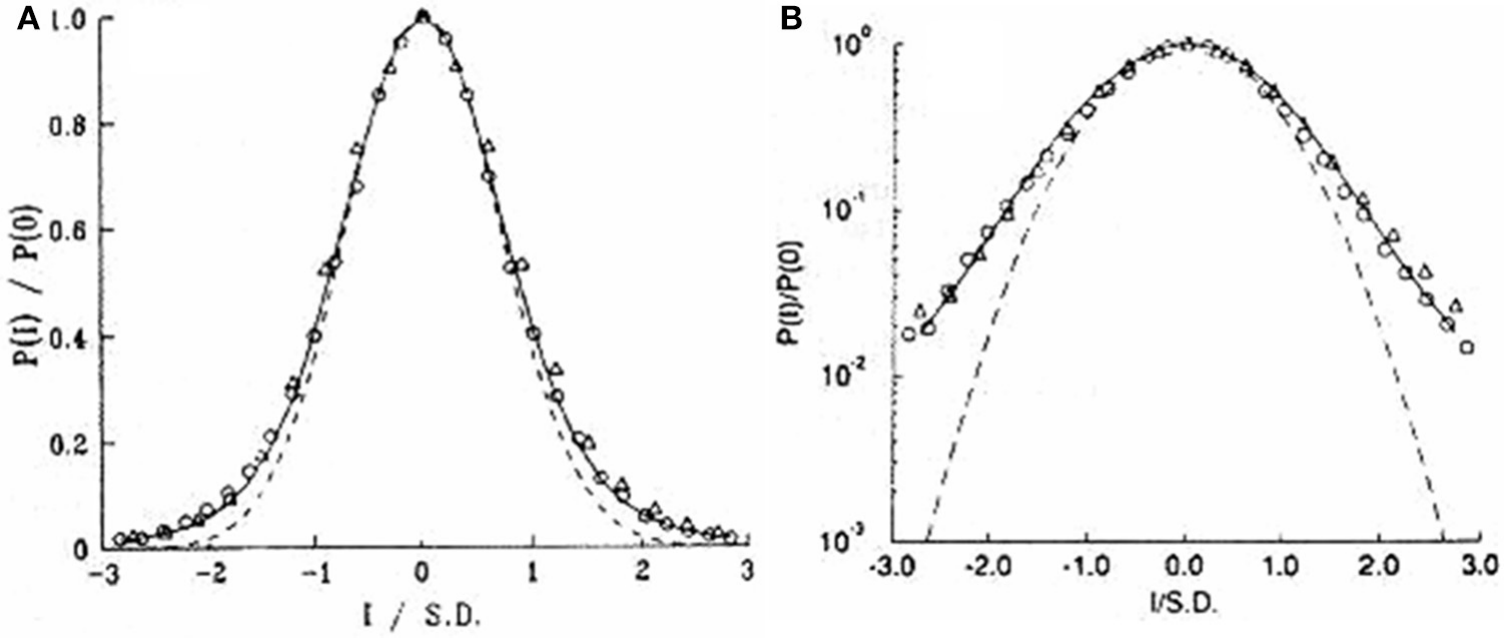
**The HRV increment data is fit with a Lévy distribution (solid curve) and compared with a Gauss distribution (dashed curve)**. Healthy (circles) and diseased (triangles) individuals are depicted with the data normalized to the standard deviation and the probabiltiy density to *P*(0). The same data are plotted in **(A,B)** only the vertical axis has been changed to a logarithm in the latter to emphasize the separation between the Gauss and empirical distributions (Adpated from Peng et al., 1993).

It has been over 20 years since Peng et al. (1993) determined that the successive increments in the cardiac beat-to-beat intervals of healthy subjects display scale-invariant, long-range anti-correlations (up to 10^4^ heart beats). They also determined that the histogram for the heartbeat interval increments is well described by the above Lévy stable distribution as shown in Figure 5. For a group of subjects with severe heart disease, they find that the distribution is unchanged, but the long-range correlations vanish. Therefore, the different scaling behavior in health and disease are related to the underlying cardiac dynamics.

The beat-to-beat time series are denoted *B*(*n*) for the beat number *n*. Peng et al. (1993) explain that the resulting HRV time series are non-stationary as a consequence of the competing neuroautonomic inputs. Parasympathetic stimulation decreases the firing rate of pacemaker cells in the heart's sinus node; sympathetic stimulation has the opposite effect. The competition between these two branches of the involuntary nervous system is the postulated mechanism for much of the erratic variability recorded in healthy subjects (Goldberger and West, 1987, Goldberger et al., 1990).

**Figure 6:**
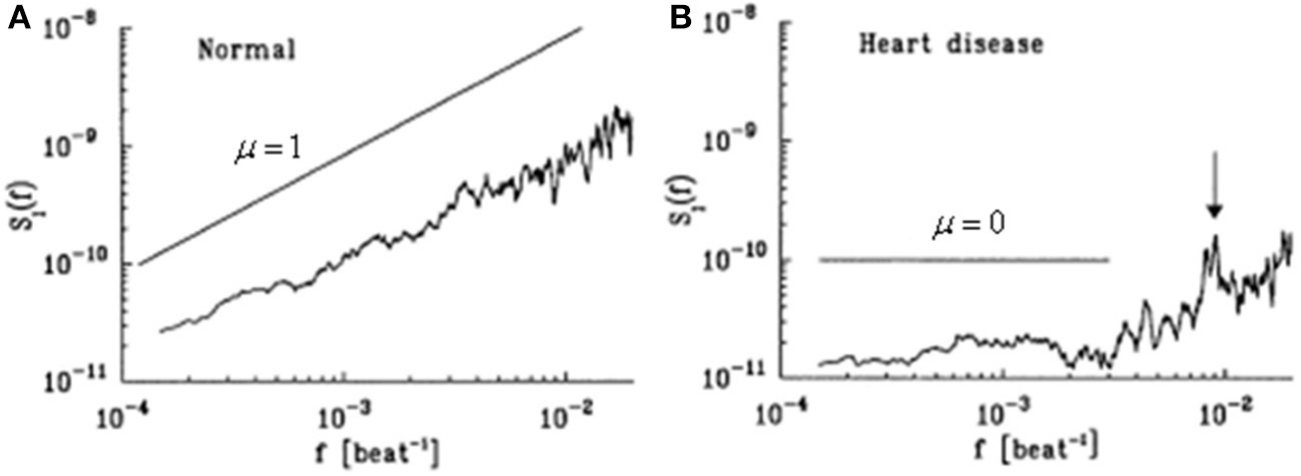
**The power spectrum for the interbeat interval increments sequences over a 24 h period. (A)** Data from healthy adult. The best-fit line for the low-frequency region has a slope 0.93. **(B)** Data from a patient with severe heart failure. The best-fit line of the low-frequency region has slope 0.14 (Adated from Peng et al., 1993 with permission).

They Peng et al. (1993), in order to remove the non-stationarity in the time series, introduced the difference in the beat interval *I*(*n*) = *B*(*n* + 1) − *B*(*n*), the interbeat increments (*x* in our notation), which they heuristically determined to be stationary. The second moment of the interbeat increment time series data scale with time as a power-law and the spectrum scales as a power law in frequency *S*(*f*) ∝ *f*^μ^ where μ = 1 − 2*H* and the mean-square level of the interbeat fluctuations increases as *n*^2*H*^, as depicted in Figure 6. Here *H* = 0.5 corresponds to Brownian motion, so that μ = 0 indicates the absence of correlations in the time series *x* (“white noise”). They observed that for a diseased data set that μ is approximately zero in the low-frequency regime confirming that the *x* are not correlated over long times. On the other hand, they also observed that for the healthy data set μ is slightly less than 1 indicating a long-time correlation in the interbeat interval differences. The anti-correlated property of *x* are consistent with a non-linear feedback system that “kicks” the heart rate away from extremes. This tendency operates on a wide range of time scales not on a beat-to-beat basis.

The spectrum indicates the correlation of the heart beats. In normal healthy individuals shown in Figure 6A the heart beats are determined to be anti-correlated. Correspondingly, this correlation vanishes in patients with heart disease (dilated cardiomyopathy) as shown in Figure 6B. This loss of correlation in diseased individuals does not influence the statistics of the heartbeat increments. Both healthy and diseased individuals are described by a Lévy stable distribution as depicted in Figure 5 and it is not possible to distinguish between the healthy and diseased individuals statistically.

More recent studies conclude that although the statistics of HRV have heavy tails they need not be strictly Lévy Hayano et al. (2011) as we discuss in Section 4. The physiologic mechanism producing the effect of keeping the heart rate away from extremes is modeled in the next section by modifying the FKE to obtain a solution that is a truncated Lévy distribution.

## 4. HRV, extrema statistics, and complexity loss

In the previous section we learned that what is of most interest in complex phenomena often resides in the tails of the PDF. Consequently, to understand complexity and its loss requires insight into the extremes of fluctuating time series. The discipline developed for the study of statistically unusual or rare events is extreme value theory, which is the branch of statistics that deals with extreme deviations from the median of the probability density. Half the data is greater than the median and half is less than the median and consequently the median is not the same as the mean or average except when the PDF is symmetric as in the case of the Normal PDF. The description of many complex phenomena has involved replacing the Normal with an IPL PDF, in fact, this replacement is often used to define complexity. The existence of the long tail implies that there are a great many more large magnitude events than in exponential processes.

The importance of rare events in statistical processes cannot be over stated since extrema dominate such processes in general, for example, in determining mortality in heart beat irregularities. A data processing model for sudden cardiac death after atrial myocardial infarction (AMI) based on a measure of the degree of non-Gaussianity of HRV has been proposed (Kiyono et al., 2006). One measure of the degree of deviation from normalcy that has been found useful in the study of HRV was developed in the study of the intermittency properties of turbulent velocity fluctuations by Castaing et al. (1990).

The analysis of the variability of RR-intervals for healthy individuals and those with heart disease were shown to have the same statistical distribution, that being Lévy stable (Peng et al., 1993). This non-Gaussian behavior of the HRV intermittent time series has, a quarter century later, been shown to be more subtle than originally believed. The scale invariant fluctuations in the healthy human heart beat were examined under a variety of statistical assumptions by Kiyono et al. (2006). They found that a truncated Lévy PDF could not be ruled out as a proper descriptor of the HRV statistics. The processing was done by aggregating the RR time series data using increasingly longer segments of the time series and eventually converged on a Gaussian distribution for sufficient coarse graining of the time series. Kiyono et al. (2006) found it impossible to distinguish between a truncated Lévy PDF and the approximated PDF based on the analysis using the technique of Castaing et al. (1990). On the other hand, the cascading mechanism that is used in the interpretation of the intermittency of velocity fluctuation in turbulent fluid flow by Castaing et al. (1990) and subsequently used in the interpretation of intermittency in HRV interval statistics could not be confirmed in the analysis of HRV by Kiyono and Bekki (2011). This contradicts an earlier finding by Lin and Hughson (2001) who found strong evidence that the cascade mechanism can generate some of the statistics of HRV variability.

**Figure 7:**
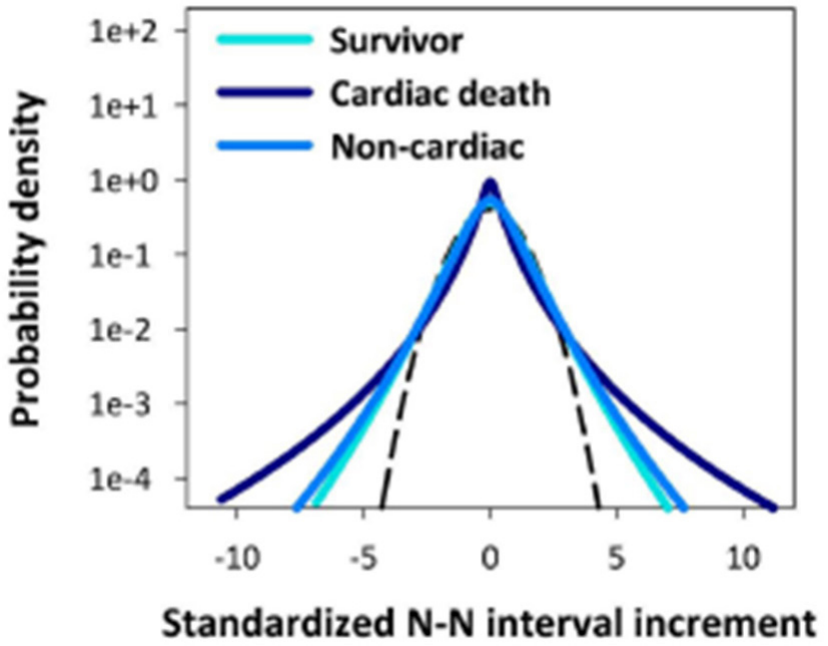
**The HRV distributions are indicated schematically from 24 h RR interval time series for a group having suffered atrial myocardial infarcation**. The patients are separated into those that suffer cardiac death, another with non-cariac death and third consisting of survivors (Adapted from Hayano et al., 2011).

In Figure 7 a qualitative sketch of the curves denote the HRV statistical PDF's from a study (Hayano et al., 2011) of a collection of 670 post-AMI (acute myocardial infarction) patients using 24 h Holter monitor data sets yielding heart beat interval variability *X*(*t*) from the time series. In this study a number of individuals suffered a cardiac death, others died by non-cardiac causes and some survived. The statistical distributions determined by the time series for the three groups are indicated schematically.

The first thing to notice about the results in Figure 7 is that even though the PDF's are only sketched it is clear that no group has Normal statistics, which in terms of the standardized variable would coincide with the dashed curve. Next the survivors and those succumbing to non-cardiac death have essentially the same variability distribution, consistent with the findings of Peng et al. (1993). The extrema for these processes would be called black swans by Taleb (2007) and are unpredictable because they share the statistics of their smaller siblings. The extrema for those that suffer cardiac death fall into the category of Sornette's dragon kings (Sornette and Johansen, 1997) and are well fit by a Lévy distribution (no truncation).

The variability statistics of the cardiac death patients are very different from those that survive even though there is a great deal of overlap in the central regions of the PDF's. The difference between the two curves suggests that those that survive might be modeled using a truncated Lévy distribution. This is consistent with the results of Kiyono et al. (2006) who find it: “..impossible to distinguish between the truncated Lévy distribution and the approximated probability density function based on Castaing's equation.”

We hypothesize a feedback mechanism that produces a truncated Lévy distribution for those that survive, not unlike the “kicks” away from the extreme excursions postulated a quarter century ago by Peng et al. (1993) to explain the anti-correlation of HRV data. This mechanism would suppress the largest extrema that persist in the cardiac death group. The pathology of the HRV PDF's being Lévy stable would then be the result of the suppression of a physiological control process, that is, a process not to inhibit events in chronological time but to suppress the size of the interbeat interval. The mathematical form of this hypothesized physiologic mechanism is given in the next subsection.

### 4.1. Truncated Lévy hypothesis

In Section 3.3 we determined that the solution to the FKE with α = 1 is the alpha-stable Lévy distribution given by Equation (33). It was noted that such distributions have fat or IPL tails that decay more slowly than the typical exponential. Such fat tails can generate diverging variances, which are not plausible for healthy physiologic data. Consequently, it is necessary to find a PDF that behaves as an IPL for intermediate amplitudes but manifests physiologic control to mitigate the occurrence of extreme events such as dragon kings. For this reason we assume that the HRV statistics of healthy individuals are determined by a physiologic feedback mechanism in which the tails of a Lévy distribution are truncated.

We hypothesize physiologic feedback to produce an exponential suppression of very large fluctuations. This exponential decay of large fluctuations can be formally incorporated into the anomalous diffusion equation Equation (6) in the following way

(36) $$\frac{\partial P(x,t)}{\partial t}=K_{\beta }ℜ{\left[\partial_{\left|x\right|}+i\gamma \right]}^{\beta }\left[P(x,t)\right].$$

The solution to the modified FKE is again given by means of Fourier transforms. The Fourier transform of the shifted operator is determined by the binomial expansion in Appendix 6.2 resulting in the equation for the characteristic function

(37) $$\frac{\partial \ln\;\tilde{P}(k,t)}{\partial t}=-K_{\beta }{\left[k^{2}+\gamma^{2}\right]}^{\beta /2}\cos\beta \left[\tan^{-1}\left(\gamma /\left|k\right|\right)\right]$$

Integrating Equation (37) yields the characteristic function after including a term to insure proper normalization of the PDF, that is, $\tilde{P}$(*k* = 0, *t*) = 1, and introducing the phase ϕ for the inverse tangent

(38) $$\ln\;\tilde{P}(k,t)=-K_{\beta }t\left\{{\left[k^{2}+\gamma^{2}\right]}^{\beta /2}\cos\beta \phi -\gamma^{\beta }\right\}.$$

The inverse Fourier transform of Equation (38) yields the truncated symmetric Lévy PDF

(39) $$P(x,t;\gamma ,\beta )=e^{-\gamma \left|x\right|}L_{\beta }\left(x,K_{\beta }t\right).$$

The truncated Lévy PDF was first studied numerically in the context of stock market fluctuations by Mantegan and Stanley (1994) and soon thereafter Koponen (1995) provided a formal derivation of the characteristic function for a truncated Lévy flight. Matshshita et al. (2003) explain that the resulting process is infinitely divisible by scaling *x* and γ with (*K*_β_*t*)^1/β^. Consequently, in terms of the scaled variables *x*_*s*_ = *x*/(*K*_β_*t*)^1/β^ and the scaled parameter γ_*s*_ = γ(*K*_β_*t*)^1/β^ the probability that an RR interval occurs in the interval (*x*, *x* + *dx*) is

(40) $$P(x,t;\gamma ,\beta )dx=e^{-\gamma_{s}\left|x_{s}\right|}L_{\beta }\left(x_{s}\right)dx_{s}$$

where *L*_β_(*x_s_*) is the stable Lévy PDF with Lévy index β.

### 4.2. Loss of complexity

It has been hypothesized that disease is associated with the loss of complexity (Goldberger et al., 1990). This hypothesis has been repeatedly tested using HRV data. For example, a study of combat casualties in an emergency department in Iraq involving 70 acutely injured adults determined that the complexity of HRV dynamics over a range of time scales was lower in high-risk than in low-risk combat casualties (Cancio et al., 2013) using a multiscale entropy (MSE) as a measure of complexity (Costa et al., 2002). In this last reference Costa et al. (2002) show that MSE uses the same coarse graining procedure that was implemented to obtain the relative dispersion in Figure 3. The scaling of the data established that the MSE was higher for healthy subjects than for those with congestive heart failure or with atrial fibrillation. It should be noted however that the loss of complexity only became evident after a certain level of coarse graining was carried out.

Consequently, in contradiction to the central limit theorem these statistical fluctuations do not converge to a Gaussian distribution. Struzik et al. (2008) emphasize an interpretation in which healthy-heart rate represents the upper bound on HRV, and reduced variability of heart rate fluctuations is of clinical risk. They call into question the complexity paradigm and its clinical interpretation. In particular they find that there is an increase in fluctuations and in complexity of heart rate in chronic-heart failure patients, in particular those at risk of death, just as those observed in Figure 7.

A quarter century ago we (Goldberger et al., 1990) proposed that the scaling behavior of the statistics may provide a measure of the complexity of the underlying process. Thus, it would appear from Figure 7 that complexity has increased in those patients that are the more severely diseased rather than the other way around as hypothesized. This interpretation proposes that complexity is proportional to variability and therefore is greatest for a non-ergodic statistical process with diverging central moments. A more thoughtful analysis of complexity reveals something different.

First of all the scaling of the statistics for the central or Lévy part of the PDF is the same for both sets of curves. However, there is the additional scaling of the truncation parameter in the truncated Lévy PDF that reduces the expanse of the fluctuations for those that do not expire by cardiac death. This second scaling, a scaling that would be produced by a control mechanism, certainly adds to the overall complexity of the process. It is the loss of this control that enables the dragon kings of cardiac death. So that although those that expire due to cardiac death have greater variability they do not have greater complexity.

The question of how to distinguish between the extrema generated by the Lévy and the truncated Lévy PDF's can be answered using extreme value theory. In Appendix 6.3 type-I and type-II extrema are discussed. Type-I extrema, those represented by a Gumbel PDF Equation (63), are generated by underlying processes with normal, log-normal, Poisson or Weibull statistics; all of which have a finite variance. Thus, we would expect that extrema generated by the truncated Lévy PDF to be attracted to the type-I PDF, since the exponential asymptotically dominates in the truncated Lévy. On the other hand, type-II extrema, those represented by a Fréchet PDF Equation (67), are generated by process with diverging second moments such as in the case of IPL's and Lévy PDF's. Thus, we hypothesize that black swans are more like type-I extrema and dragon kings are more like type-II extrema. So how readily can we distinguish between the two?

#### 4.2.1. Extrema statistics

Leonard Tippet (1902–1985) was the founder of the field of extreme value statistics. He was employed by the British Cotton Industry Research Association, where he worked on understanding how to make cotton thread stronger. In his studies, he realized that the strength of a thread was controlled by the strength of its weakest fibers. He determined that perhaps the most intuitive comparison between the extrema PDF's is given by the return time, that is, after a given magnitude event occurs how long must we wait for another event of that magnitude to occur? In this way the statistics of the size of an event can be related to the variability of the size over time.

**Figure 8:**
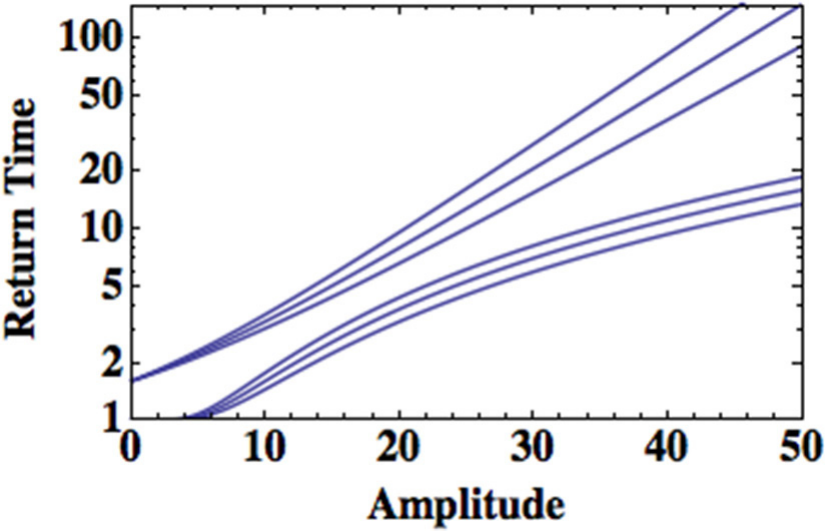
**The return time is plotted as a function of the amplitude of the extrema for type-I and type-II extrema**. The upper curve uses the Gumbel distribution and the lower curve uses the Fréchet distribution in Equation (41). The parameter values have been selected with ± 10% variation to contrast the two distributions.

Recall that the AMI cohort group introduced earlier was defined by having had an initial AMI event. So the question arises as to how long we expect a person to wait before the occurrence of a second event. This can be framed as a return time problem and depends on whether an individual's HRV is determined by a Lévy or by a truncated Lévy PDF. This is the kind of question encountered in the consideration of earthquakes and floods. The return time to an extreme value η is in general given by

(41) $$T\left(\eta \right)=\frac{1}{1-F(\eta )}$$

where *F*(η) is the probability of achieving this extreme value. Figure 8 displays the return time using the Gumbel PDF, which is a consequence of the underlying process being a truncated Lévy, as well as the Fréchet PDF, which results from the underlying processes being Lé vy stable, Mittag-Leffler or a Pareto PDF. The schematic behavior of the return time is depicted for typical parameter values using Equation (41). In this figure it is clear that the return times for a phenomenon with a diverging variance is significantly shorter than for one with a finite variance.

For a Gumbel PDF a magnitude “40” event (arbitrary units) occurs approximately every 50 units of time, say years for an earthquake, but for a Fréchet distribution this event occurs approximately every 10 units of time or so; a factor of five more frequently. The return time increase for type-I extrema is approximately exponential with extrema size (amplitude). On the other hand, the return time increase for type-II extrema parallels the type-I for small values of the amplitude but seems to approach a saturation level at large extrema. Consequently the return time for type-I extrema diverges from that of type-II extrema with increasing size. It is not clear that it is easier to identify what needs to be done to develop protection against the rare events of a truncated Lévy process (type-I extrema) than for the significantly more frequent Lévy process (type-II extrema). However, the relative frequency of the two classes of extrema would suggest very different strategies for suppressing them, particularly in identifying and fixing a disrupted control process that results in the loss of truncation resulting in Lévy distributed HRV.

#### 4.2.2. Predicting survival

A second example of a physiologic phenomenon that is asymptotically described by IPL variability is epileptic seizure. Recently Osorio et al. (2010) presented a dynamic analogy relating seizures, called “brainquakes,” and earthquakes. They supported the analogy using five scale-free statistics: the Gutenberg-Richter distribution of event intervals, the Omori and inverse Omori laws, and the conditional waiting time until the next event. Somewhat earlier Beggs and Plenz (2003) had found that the brain produced “neuronal avalanches,” which like the brainquakes have no characteristic scale and in addition their PDF is an IPL with index −3/2. This and related work was reviewed by Osorio et al. (2010) to emphasize that the purported resemblance between earthquakes and neuronal spiking had not been previously subjected to the kind of rigorous scrutiny presented in their paper. They point out that the behavior of neuronal assemblies and of epileptogenic regions of the brain are fractal and the observable changes in neuronal voltage is self-similar.

What is perhaps most significant in the discussion of brainquakes for our purposes is the observation first made by Davies et al. (1989) regarding interquake intervals for fat-tailed distributions. They asked the question:

Is it true that, “The longer it has been since the last event, the longer the expected time till the next?.”

This means that the average waiting time conditional on the time elapsed since the last event *t* denoted by 〈τ |*t*〉 increases with *t*. Sornette and Johansen (1997) provide an analytic answer to this question and we follow their argument in outline below.

The probability that an event occurs in the time interval (*t*, *t* + *dt*) is denoted as ψ(*t*) *dt* given that the last quake occurred at time *t* = 0. The waiting-time PDF is given by ψ(*t*) so that the probability that no event has occurred in the time interval (0, *t*), the survival probability, is given by

(42) $$\Psi \left(t\right)=\underset{t}{\int^{\infty }}\psi \left(t^{\prime }\right)dt^{\prime }=1-\underset{0}{\int^{\infty }}\psi \left(t^{\prime }\right)dt^{\prime }.$$

These two distributions can be used to define *P*(τ |*t*)*d*τ the probability that the next event will occur in the time interval (*t* + τ, *t* + τ + *d*τ) conditional on the fact that the last quake had not occurred up to the time *t*. The formal expression for this probability density is given by Bayes' theorem to be (Sornette and Johansen, 1997):

(43) $$P(\tau |t)=\frac{\psi \left(t+\tau \right)}{\Psi \left(t\right)}$$

so the conditional average waiting time is determined by the integral

(44) $$\langle \tau |t\rangle =\overset{\infty }{\int_{0}}\tau P(\tau |t)d\tau .$$

The asymptotic form of the Lévy and Mittag-Leffler PDF's discussed as solutions to the KFE are IPL's, which is to say, they are Pareto PDF's, so for the present discussion we consider the schematic form for the waiting-time PDF

(45) $$\psi \left(t\right)=\frac{\mu A}{t^{\mu +1}}$$

where *A* is a constant. Evaluating the survival probability corresponding to the IPL waiting-time PDF yields for the conditional PDF Equation (43)

(46) $$P(\tau |t)=\frac{\mu }{t}{\left(\frac{t}{t+\tau }\right)}^{\mu +1}$$

which when inserted into Equation (44) allows us to determine the conditional expected time to the next quake for μ > 1

(47) 〈τ|t〉∝t

It is evident that for an inverse power-law waiting-time PDF the conditional expected waiting time to the next quake increases linearly with the elapsed time since the last quake. Consequently, the longer it has been since the last quake the larger is the conditional expected waiting time.

**Figure 9:**
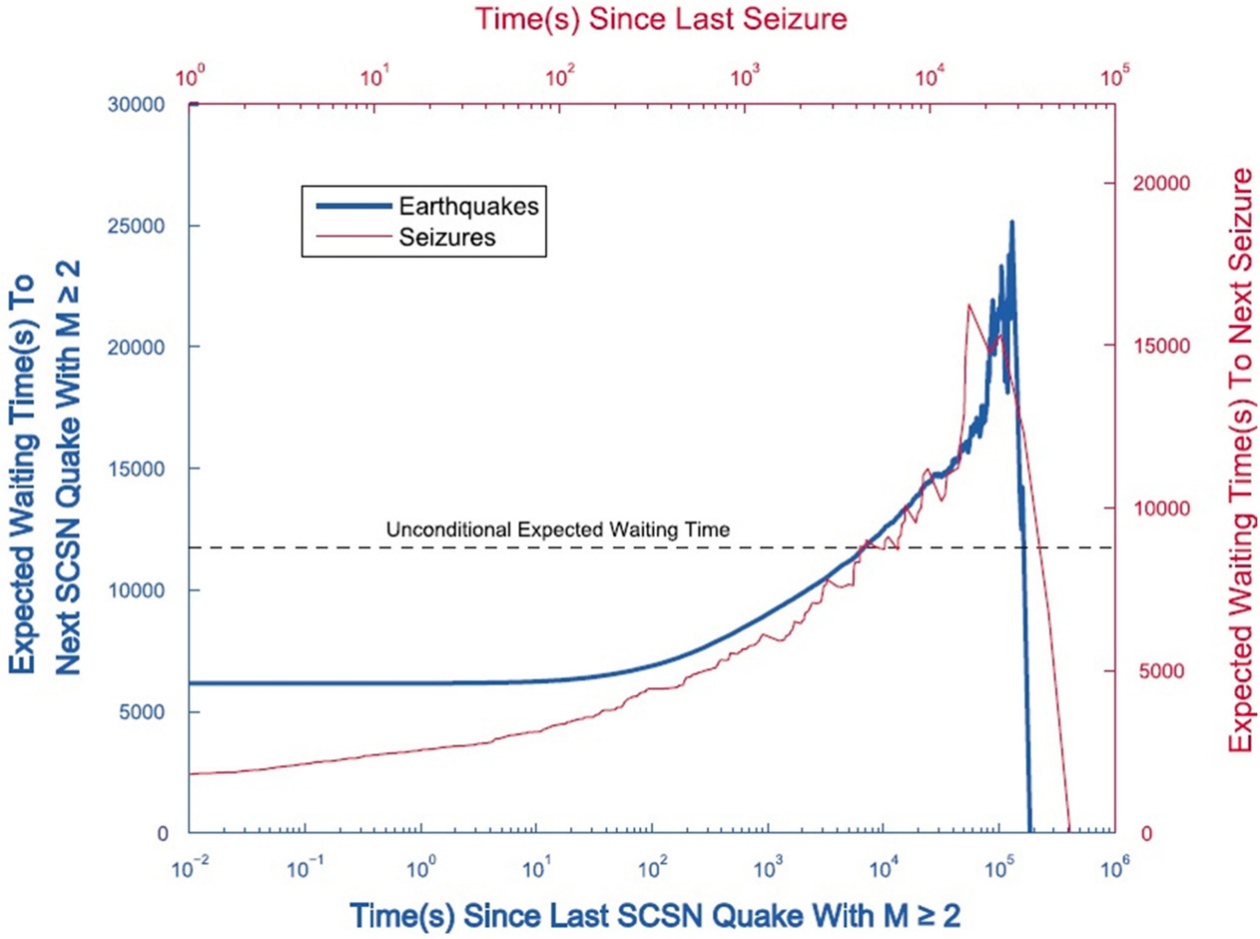
**Average conditional waiting time 〈τ |*t*〉 until the next event conditional on the the time *t* already elapsed since the last event ended**. Seizures (thin curve; upper x axis and right y axis); earthquakes (thick curve; lower x axis and left y axis). For seizures and earthquakes, 〈τ |*t*〉 increases paradoxically with up to a maximum (due to finite-size effects). The dashed horizontal line shows the value of the unconditioned average waiting time between two events. The increase in 〈τ |*t*〉 with *t* confirm the heavy-tailed nature of the distribution of interevent times. M, magnitude. From Osorio et al. (2010) with permission.

Figure 9 depicts the conditional waiting time as a function of the time since the last event for both seizures and earthquakes (Osorio et al., 2010). The dashed line is the unconditioned expected waiting time calculated for an underlying exponential PDF. At early times the conditional expected waiting times are less than the unconditioned value, however at late times, when the asymptotic IPL is expected to be valid, they exceed this value. Thus, at late time the question asked by Davies is answered in the affirmative.

Sornette and Johansen (1997) determine the general conditions under which the question can be answered both positively and negatively. They summarize their finding with the observation that with the exception of the Poisson PDF all statistical descriptions must have a conditional average time from now to the next event that depends on the time since the last event. Whether the question is answered positively or negatively depends on whether the waiting-time PDF falls off at a rate slower or faster than the exponential, respectively. The exponential is neutral with respect to the question because the time since the last event has no influence on the time of the next event and consequently establishes the statistical crossover between the two states. Huillet and Raynaud (2001) re-examine the question and generalize the analysis to renewal events. They find that the question should be replaced with:

Is it true that: “The longer it has been since the last earthquake, the longer the median time till the next.”

Note that “expected time” in the original question is replaced with “median time” in the new question since in the analysis the median time is always finite whereas the expected time may in fact diverge.

#### 4.2.3. Age-related disease (ARD) networks

Medicine concentrates on keeping us healthy, and extending our life expectancy through the maintenance of the complexity of the human body. However, we all die in the end. Therefore, we have a vested interest in determining if science can find ways to facilitate life extension. Cluett and Melzer (2009) mention that ageing has been described as the result of the lifelong accumulation of random molecular damage, which in turn depends of the balance between molecular damage and repair. They review how ARD's are associated with gene variants and how these variants determine pathways linked to ageing, what they call “beacons.”

Budovsky et al. (2007) were the first to apply networking ideas to the study of longevity, focusing on the analysis of human protein-protein interactions (PPI's), which they call the “longevity network.” They point out that the longevity gene-encoded proteins together with their interacting proteins form a continuous scale-free network with an extremely large number of hubs. The hubs are significant since, as they point out, almost all of them are involved in at least one ARD. Note that this is the topological complexity referred to earlier as being separate and distinct from the temporal complexity (West et al., 2014) discussed in relation to physiologic time series.

Continuing in this departure from the trend of focusing on individual genes and/or their products Wolfson et al. (2009) emphasize that the properties of complex networks cannot be reduced to the properties of its components, and protein networks are no exception. They showed that longevity-associated proteins (LAP's) or ARD proteins can be organized into scale-free network as identified in human cancer proteins, proteins involved in Alzheimer's disease, type 2 diabetes, and atherosclerosis (Budovsky et al., 2007). The increased incidence of ARD's in advanced age is considered a major factor limiting human lifespan (Cutler and Mattson, 2006).

In this context TNE would support the hypothesis that ARD's and longevity networks are interconnected through the signaling of the proteins on the network and that such signaling can be associated with the control of lifespan (Warner, 2005). In addition the application of the network perspective to biogerontology has been carried out by Tacutu et al. (2012) to identify new “putative regulators of longevity” and as commented by one referee, this indicates a high functional significance of TNE.

## 5. Discussion and conclusions

It is often the case in presenting mathematical arguments in support of a clinical interpretation that the latter is lost in the torturous details of the former. So let us recap the most important aspects of the formal discussion. First of all the statistics most often observed with time series generated by physiological phenomena, both healthy and diseased, are fractal and therefore they scale. This scaling suggests the hypothesis that the behavior of the PDF is described by a FKE. In general the fractal dimensions associated with the scaling of the FKE are complex, they have a real and imaginary part, with the real part denoting the index for the IPL and the imaginary part determining the period of a log-periodic modulation of the statistics.

One solution to the FKE was the Mittag-Leffler PDF and another was the Lévy stable PDF, both of which have a Pareto IPL form asymptotically. A generalization of the FKE that incorporated a physiological control mechanism to suppress large-scale fluctuations yields a truncated Lévy PDF. The truncated distribution was associated with a healthy HRV time series, whereas the Lévy PDF was associated with the statistics for a diseased HRV time series. Since the variability for a Lévy process is greater than for its truncated counterpart this seemed to contradict the hypothesis that a healthy physiologic process is more complex than a diseased one. This is not the case.

A sharper definition of physiologic complexity is needed. In the *Scientific American* article (Goldberger et al., 1990) the mistaken conclusion could have been drawn that complexity and variability were the same thing. This is not always the case. Consider, for example, a generalized hyperbolic, a Lévy or a Mittag-Leffler PDF. They each have a Pareto PDF asymptotically, however their level of complexity is not the same. The generalized hyperbolic and Mittag-Leffler PDF's do not scale throughout the domain of the variate, but only asymptotically. The Lévy PDF, on the other hand, is infinitely divisible and scales throughout the domain of the variate. This would imply that in the former case the complexity associated with the bursting behavior of scaling time series is only evident for very long time series and would not be observed in short time series. This kind of complexity is revealed by the renormalization group behavior observed in the discussion of the dragon king.

This partitioning into the simple and the complex was made evident in an argument put forward by Montroll and Shlesinger (1982) in which the random fluctuations of an economic time series is described by a function *g*(*z*) that has finite central moments. A process was generated in which mechanisms were introduced that scaled in time with increasing values *b*, *b*^2^,.. with probabilities *a*, *a*^2^,.., respectively, resulting in the renormalization group relation for the new PDF

(48) G(z)=g(z)+aG(bz).

They used this argument to derive the Pareto IPL form for the PDF of income for the highest few percent of income earners in Western countries independently of the form of *g*(*z*) as long as it has finite central moments. As stated in the text this argument was applied to the scaling of the bronchial airway network in the mammalian lung (West et al., 1986, Shlesinger and West, 1991) with income level replaced with the branching generation. The resulting distribution is *g*(*z*) for small values of the argument but having an IPL (Pareto) form asymptotically with index μ = log *a*/log *b*.

Consequently, the level of complexity is a balance between regularity and variability, not just the result of variability alone. Thus, the truncated Lévy PDF has this balance built in with the possibility of extreme variability at short time intervals, but suppressed extreme variability at very long time intervals. In keeping with this new perspective, homeostasis, which focuses on the long time control of a physiologic process, and inverse power laws that focus on unfettered variability each have only part of the answer. TNE is intended to capture the full range of complexity characteristic of physiological phenomena that have evolved through the development of complex dynamic networks.

Herein we have stressed that the TNE hypothesis encompasses the fact that the statistics of physiologic processes are not the Normal statistics of simple physical systems. Instead complex physiological phenomena are dominated by IPL's of various forms. The scaling of physiologic time series data manifest in the IPL's strongly suggest that the equations of motion for the PDF describing this behavior have fractional derivatives in the variate, in time or both. The solutions to the FKE's have this IPL structure as evidenced by the asymptotic behavior of both the Lévy and Mittag-Leffler PDF's. A generalization of the FKE, under the additional hypothesis that there exists a physiological mechanism to inhibit large excursions of physiological fluctuations, was shown to produce a truncated Lévy PDF. Although it was not shown here a similar generalization can be made to generate a truncated Mittag-Leffler function.

We have argued that a dragon king extreme (generated by a Lévy stable PDF) is much more likely to occur in a given interval of time for a physiologic process than a black swan extreme (generated by a truncated Lévy stable PDF). Consequently, if the extreme event is medically critical a person is more likely to die as the result of a dragon king than of a black swan. However, if we can identify the physiological mechanism that produces the truncation in the Lévy statistics, the one whose suppression transforms an unpredictable black swan into a more tractable dragon king, then a judicious intervention could make a person less vulnerable to the more frequent dragon kings.

The existence of an explicit physiological mechanism that suppresses large scale excursions of heart beat intervals has been hypothesized. Such a mechanism would seem to explain the clinical data and to be consistent with the fractional calculus developed in this paper as a way of systematically describing the dynamics of HRV for both the healthy and those with a variety of heart diseases. Moreover it is not unlikely that such a mechanism is not process specific but is a natural consequence of biological evolution. It has been shown elsewhere that fractal statistics confer a survival advantage (West, 2006) and therefore it would not be surprising if nature adopted a generic mechanism to limit the range of such fractal fluctuations while simultaneously retaining the benefits such scaling provides.

We close with a speculation having to do with the relatively flat region of the conditional waiting time for an event observed in Figure 9. This behavior indicates a lack of dependence of the conditional waiting time on the elapsed time since the last event for short times and is followed by the linear dependence suggested by the IPL PDF at late times resulting from TNE. Although not established here this pattern is consistent with the qualitative behavior of the underlying statistics being described by a Lévy PDF. One may speculate that although dragon kings have a shorter unconditional return time than do black swans based on their amplitude that counterintuitively for a given elapsed time since the last event the conditional expected time until the next dragon king is longer than the unconditioned average waiting time. By the same token the conditional expected time until the next black swan is shorter than the unconditioned average waiting time. Thus, one consequence of TNE is the fact that although the recurrence time between extreme events is shorter for Type-II over Type-I statistics, the conditional time between extreme events increases with the time since the last event.

### Conflict of interest statement

The author declares that the research was conducted in the absence of any commercial or financial relationships that could be construed as a potential conflict of interest.
