# Supplementary material for: A mathematics for medicine: The Network Effect
Source: Front Physiol. 2014 Dec 9;5:456. doi: 10.3389/fphys.2014.00456 (PMC4260484; doi:10.3389/fphys.2014.00456)
Supplement: Supplementary file 1 [file Presentation1.PDF]

1 Appendices

1.1 Glossary

allometric control: A control process that makes use of the interconnectedness across the scales contributing to an IPL distribution. An IPL dynamic network would require an allometric control process to simultaneously control the span of time scales contributing to the dynamics.

anomalous diffusion: Classical diffusion has a variance that increase linearly with time. The diffusion is termed anomalous when that increase in time has a power-law index different from one.

black swan: A typical statistical distribution has a range of values for the fluctuating variable. Those values that deviate an exceptionally large distance from the mean were identified as black swans by Taleb [7]. These entities are usually discussed in the context of inverse power-law and not Normal statistics.

chaos: The solution to a nonlinear differential equation has a sensitive dependence on the initial state of the system. The sensitivity is so great that any change in the initial state, no matter how small, will eventually lead to a totally different final state; one that could not be predicted from the original initial state. In many cases this is observed as a trajectory that is rich in variability, a so-called fractal trajectory (see below).

dragon king: This term was introduced by Sornette [6] to distinguish those rare events whose statistics differ from those of the majority of the data in which they are found. It is this change in statistics that distinguishes dragon kings from black swans and often suggests ways to predict them.

extrema: The fluctuations in a statistical process that exceed some prescribed, and typically large, value.

extrema statistics: The statistics of extrema are very different from the statistics of the process from which they are drawn. For example, all exponential statistical processes have Gumbel distributions for their extrema; whereas all IPL statistical processes have Fréchet distributions for their extrema.

fixed point equation: A mathematical term meaning a function that maps onto itself. Consequently, iterating a process by repeated application of the same function, will not change its value at a fixed point.

fractional calculus: The ordinary calculus with integer derivatives and partial derivatives is only one of many calculi, just as Euclidean geometry is only one of a number of different geometries. The calculus with non-integer derivatives and non-integer partial derivatives is over three hundred year old, but has only found wide-spread application in science and biomedicine in the last decade as a way to understand complexity.

Fokker-Planck equation FPE); The equation of evolution for a probability density function in phase space that is first-order in time and second-order in space. The solution to this equation describes how an ensemble of random variables changes over time, for example, the diffusive spreading of dye in a fluid.

fractional Fokker-Planck equation (FFPE): The equation of evolution

for a probability density function in phase space that is fractional-order in time and/or in space, with the fractional indices not being the same in general. The solution to this equation is typically IPL asymptotically.

fractals: A fractal structure is not smooth and homogeneous, but rather when examined with stronger and stronger lenses, reveals greater and greater levels of detail such as in the structure of bronchial airways and in the cardiac conduction network. A fractal statistical process is one in which there is a statistical rather than geometrical sameness to the process at increasing levels of magnification. This is observed in the fluctuations of HRV time series; a fractal time series.

fractal dimension: One measure of a fractal object is its dimension, which is non-integer. The fractal dimension measures the degree of irregularity of an object such as the foldings of the surface of a mammalian brain or the branching structure of bronchial airways [10]. The fractal dimension can have an imaginary component that captures a log-periodic aspect of the underlying process.

inverse power law (IPL): The probability that a random variable X has a value in an interval $(x, x + dx)$ is $p(x)dx$ and the probability density function (PDF) is IPL when $p(x) \propto 1/|x|^{\alpha}$. The IPL index is α and for $\alpha < 2$ the second moment diverges.

Lévy statistics: There is no simple analytic expression for the PDF describing such statistics in general although special cases do exist. The defining property has to do with joining data sets. If two data sets having Lévy statistics are joined the resulting data set also has Lévy statistics. These are called alpha-stable Lévy processes and they are the limit distributions that satisfy a generalization of the central limit theorem to data with diverging second moment. A truncated Lévy process is one for which the long tails are suppressed by an exponential.

nonlinear dynamics: The equations of motion for most dynamic systems have a dependence on the dynamic variable raised to a power greater than one. The solutions to such equations have been shown to be generically unstable and the specific form of the instability is now called chaos. In a network context the nonlinear dynamics have been shown to result in criticality, which depending on the application has been called cooperative behavior, consensus or phase transitions [11].

Pareto distribution: The first empirical inverse power-law distribution was obtain for the distribution of income by Pareto and now bears his name.

phase space: Such a space has axes given by the possible values of the dynamic variables specifying the system of interest. The evolution of a system is recorded by a curve in this space indexed by the time variable.

renormalization group: A function $Z(t)$ is a member of a renormalization group when scaling the time t by a parameter λ yields $Z(\lambda t) = \lambda^{\alpha} Z(t)$ and α is a scaling index. Equations of this form have a solution that is power law in time with an index that can be positive or negative.

scaling: Typically this refers to the process by which a renormalization group equation is obtained. It implies that the various scales contributing to dynamic process are coupled together as a power law through the index α.

self-similarity: This concept was the precursor to the scaling observed in fractals and also involves the replication of a given structure at successive scales. It has been used to describe the organization of structure of the mammalian lung at successive generations, but it fails to take into account the variability of the airways within a given generation that is accounted for in the fractal lung model [8].

stochastic equations: The equations of motion for a process whose time variation is determined at least partly by random fluctuations in time. Therefore the traditional differential equations of motion have to be reinterpreted to properly account for the statistical nature of the driver. There are a number of stochastic calculi just as there are a number of fractional calculi and geometries.

subordination: This is a process whereby the behavior of one process is subordinated to that of another process. In probability theory it has a technical meaning involving the transformation from one variable to another. In biology the time measured by the clock can be very different from the time experienced by an organism and the two have been interrelated by means an allometry relation [9], for example, a 'normal' person and one addicted to drugs experience time in measurably different ways.

Tauberian theorem: This is handy mathematical relations between a power law in a variable $t^{\alpha-1}$ and its Fourier or Laplace transform variable $1/s^{\alpha}$. The utility of this theorem should be evident for the discussion of IPL PDF's.

1.2 The shifted Reisz fractional derivative

The modified anomalous diffusion equation introduces the shifted Reisz fractional derivative, which in this Appendix we evaluate using a binomial expansion of the operator. Using the definition of the Reisz fractional derivative [4]:

$$\partial_{|x|}^{\kappa}\left[g(x)\right] \equiv \Gamma\left(\kappa-1\right)\frac{\sin\left(\kappa\pi/2\right)}{\pi}\int_{0}^{\infty}d\xi\ \xi^{1-\kappa}\left\{g^{''}\left(x+\xi\right)+g^{''}\left(x-\xi\right)\right\} \qquad (1)$$

where the prime denote derivatives with respect to x. In the text we wish to take the Fourier transform of the shifted operator so we substitute $g(x)=e^{ikx}$ into Eq.(1) to obtain

$$\partial_{|x|}^{\kappa}\left[e^{ikx}\right] \equiv -k^{2}e^{ikx}\Gamma\left(\kappa-1\right)\frac{\sin\left(\kappa\pi/2\right)}{\pi}\int_{-\infty}^{\infty}d\xi\ \left|\xi\right|^{1-\kappa}e^{ik\xi}. \qquad (2)$$

The integral is given by [2]

$$\int_{-\infty}^{\infty}d\xi\ \left|\xi\right|^{1-\kappa}e^{ik\xi}=\frac{\left|k\right|^{\kappa-2}}{2\Gamma\left(\kappa-1\right)\sin\left(\kappa\pi/2\right)};\quad \kappa\neq-1,-2,...$$

which when substituted into Eq.(2) yields

$$\partial_{|x|}^{\kappa}\left[e^{ikx}\right] = -\left|k\right|^{\kappa} e^{ikx}. \tag{3}$$

Here we see an eigenvalue relation where e^{ikx} is the eigenvector and $-\left|k\right|^{\kappa}$ is the eigenvalue.

We now apply the binomial theorem to the shifted Reisz operator interpreted as the real part of an operation

$$\mathrm{Re}\left(\partial_{|x|} + i\gamma\right)^{\beta}\left[e^{ikx}\right] = \sum_{n=0}^{\infty}\begin{pmatrix}\beta\\ n\end{pmatrix}\mathrm{Re}\left(i\gamma\right)^{n}\partial_{|x|}^{\beta-n}\left[e^{ikx}\right]. \tag{4}$$

Using the eigenvalue relation we write

$$\partial_{|x|}^{\beta-n}\left[e^{ikx}\right] = -\left|k\right|^{\beta-n} e^{ikx}$$

which enables us to write

$$\begin{aligned}\mathrm{Re}\left(\partial_{|x|} + i\gamma\right)^{\beta}\left[e^{ikx}\right] &= -\sum_{n=0}^{\infty}\begin{pmatrix}\beta\\ n\end{pmatrix}\left|k\right|^{\beta-n}\mathrm{Re}\left(i\gamma\right)^{n} e^{ikx}\\ &= -\mathrm{Re}\left\{\left(i\gamma + \left|k\right|\right)^{\beta}\right\} e^{ikx}. \end{aligned} \tag{5}$$

This last equation is obtained regardless of the ordering of the exponents in the binomial expansion as is easily checked. Eq.(5) can be further simplified using

$$Ae^{i\phi} = i\gamma + \left|k\right|$$

to obtain the amplitude and phase

$$A = \left(\gamma^{2} + k^{2}\right)^{1/2} \quad \text{and} \quad \phi = \tan^{-1}\left(\frac{\gamma}{\left|k\right|}\right)$$

which when substituted into Eq.(5) yields

$$\mathrm{Re}\left(\partial_{|x|} + i\gamma\right)^{\beta}\left[e^{ikx}\right] = -\left(\gamma^{2} + k^{2}\right)^{\beta/2}\cos\beta\phi\; e^{ikx}. \tag{6}$$

The solution given by Eq.(6) is easily checked by taking the limit as the shift parameter vanishes. In this case

$$\lim_{\gamma\to 0}\mathrm{Re}\left(\partial_{|x|} + i\gamma\right)^{\beta}\left[e^{ikx}\right] = -\left|k\right|^{\beta} e^{ikx}\lim_{\gamma\to 0}\cos\beta\left[\tan^{-1}\left(\frac{\gamma}{\left|k\right|}\right)\right]$$

and the final limiting term is unity so that

$$\lim_{\gamma\to 0}\mathrm{Re}\left(\partial_{|x|} + i\gamma\right)^{\beta}\left[e^{ikx}\right] = -\left|k\right|^{\beta} e^{ikx}$$

thereby reducing to the original eigenvalue relation Eq.(3) as it should.

1.3 Comments on Extreme Value Theory

The theory of extreme events has been most completely developed in large part for independent random variables since events that are sufficiently rare are necessarily statistically independent of one another. The following remarks on extreme statistics are taken from Lindenberg and West [5]. Consider the set of m observations $\{X_j\}$ $j = 1, .., m$ taken from a continuous process. We define the cumulative distribution

$$F(\eta, m) \equiv \Pr ob \{T(\eta) > m\Delta t\} \tag{7}$$

where Δt is the time interval between observations and $T(\eta)$ is the time when the random variable first crosses $X = \eta$ and Eq. (7) defines the probability this value is achieved after m measurements. Consequently, we can define the probability of achieving the value $X = \eta$ for the first time in the time interval $(m\Delta t, (m+1)\Delta t)$ to be

$$\Delta tp(\eta, m) = -\left[F(\eta, m+1) - F(\eta, m)\right] = \Pr ob\left\{m < \frac{T(\eta)}{\Delta t}, m+1\right\}. \tag{8}$$

Let $P(x)$ be the cumulative distribution for each of the random variables X_j:

$$P(x) \equiv \Pr ob \{X_j < x\}. \tag{9}$$

The probability $P(x)$ is viewed as a phenomenological representation of the unspecified underlying dynamical process and contains all the information that one needs to determine the extremal statistics of the data set $\{X_j\}$. thus, the cumulative distribution Eq.(7) is the joint probability that each distribution does not exceed η :

$$F(\eta, m) = [P(\eta)]^m \tag{10}$$

Inserting this expression into Eq.(8) yields for the probability of achieving η for the first time between the m and $m+1$ measurements is

$$\Delta tp(\eta, m) = [P(\eta)]^m [1 - P(\eta)]. \tag{11}$$

The mean first passage time to η in this discrete representation is given by the average

$$T_1(\eta) = \sum_{m=0}^{\infty} m\Delta tp(\eta, m) = \frac{\Delta t}{1 - P(\eta)}$$

which solving for $P(\eta)$ and inserting the value into Eq.(10) yields

$$F(\eta, m) = \left[1 - \frac{\Delta t}{T_1(\eta)}\right]^m. \tag{12}$$

In the limit $\Delta t \to 0$ and $m \to \infty$ such that their product $t = m\Delta t$ is constant Eq.(12) yields the waiting time distribution

$$F(\eta, t) = \lim_{\Delta t \to 0, m \to \infty} \left[1 - \frac{\Delta t}{T_1(\eta)}\right]^m \Rightarrow \exp\left[-\frac{t}{T_1(\eta)}\right] \tag{13}$$

which provides a measure of the rareness of the extreme value η. This exponential form for the cumulative distribution is typical for independent events.

The functional dependence of the cumulative distribution on η depends on the particular process being studied and is manifest in both the mean first passage time $T_1(\eta)$ and the probability $P(\eta)$. Lindenberg and West [5] point out that it is however possible to provide a general classification of the asymptotic behavior of the cumulative distribution Eq.(13). This can be done because the asymptotic behavior is dominated by the tails of the phenomenological distribution and is insensitive to other details of its structure. There are three general classes of asymptotic distributions and here we only consider "types I- and II- extreme values". The type-I extreme value distribution requires that $P(\eta)$ decrease at least exponentially with increasing η in the sense that $P(\eta) < \exp[-f(\eta)]$, where $f(\eta) \leq (\ln \eta)^a$ with $a > 1$. Examples of distributions $P(\eta)$ leading to this class of distributions of extrema are: normal, log-normal, Poisson and Weibull. a type-II extreme value distribution occurs when $P(\eta)$ does not possess finite central moments. For example, IPL and Lévy PDF's belong to this class.

An interesting feature of these two types of extrema distributions is that each is characterized by a universal form. Consider the asymptotic distribution

$$F(y) \equiv \lim_{t\to\infty} F(\eta, t) \tag{14}$$

where y is defined separately for the two distributions. For type-I extreme values one has the Gumbel distribution [3]

$$F(y) = \exp[-\exp(-y)] \tag{15}$$

where

$$y = \alpha_j (\eta - \beta_j) \tag{16}$$

and α_j and β_j are defined by the relations

$$P(\beta_j) = 1 - \frac{1}{j} \tag{17}$$

and

$$\alpha_j = j\frac{dP(x)}{dx}\Big|_{x=\beta_j} \ . \tag{18}$$

For type-II extrema values with $y \geq 0$ are determined by the Fréchet distribution [1]

$$F(y) = \exp\left[-y^{-\alpha}\right] \quad ; \quad \alpha > 0 \tag{19}$$

where

$$y = \frac{\eta}{v_j} \tag{20}$$

and v_j is the expected largest value in the sample of size j and is given by the relation

$$P(\beta_j) = 1 - \frac{1}{j}\left(\frac{v_j}{\eta}\right)^{\alpha} \tag{21}$$

References

[1] M. Fréchet, *Ann. de la Soc. polonaise de Math.* **6**, 93, Cracow (1927).

[2] I.M. Gel'fand and G.E. Shilov, *Generalized Functions, Vol. 1*, translated by E. Saletan, Academic Press, New York (1964).

[3] E. Gumbel, *Statistical Theory of Extreme Values and Some Practical Applications, Applied Mahtematics Series* **33**, National Bureau of Stadards (1954).

[4] R. Herrmann, "Uniqueness of the fractional derivative definition", *arXiv 1303:2939v3* (2013).

[5] K. Lindenberg and B.J. West, "The first, the biggest, and other such considerations", *J. Stat. Phys.* **42**, 201 (1986).

[6] D. Sornette, "Dragon-Kings, Black Swans and the Prediction of Crises", *Int. J. Terraspace Sci. & Eng.* **2**, 1 (2009).

[7] N.N. Taleb, *The Black Swan*: *the impact of the highly improbable*, Random House (2007).

[8] B.J. West, V. Bhargava and A.L. Goldberger, "Beyond the principle of similitude: renormalization in the bronchial tree", *J. Appl. Physiol.* **60**, 189 (1986).

[9] D. West and B.J. West, "Physiologic Time: A hypothesis", *Physics of Life reviews* **10**, 210 (2013).

[10] B.J. West, *Fractal Physiology and Chaos in Medicine*, World Scienific, Singapore (1990); Revised Second Ed. (2013)

[11] B.J. West, M. Turalska and P. Grigolini, *Network of Echoes: Imitation, Innovation and Invisible Leaders*, Springer, NY (2014).
